# Renal Denervation Effects on Blood Pressure in Resistant and Uncontrolled Hypertension: A Meta‐Analysis of Sham‐Controlled Randomized Clinical Trials

**DOI:** 10.1002/clc.70104

**Published:** 2025-03-01

**Authors:** Hamidreza Soleimani, Babak Sattartabar, Bahar Parastooei, Reza Eshraghi, Roozbeh Nazari, Soroush Najdaghi, Sara Hobaby, Ali Etemadi, Mehrdad Mahalleh, Maryam Taheri, Adrian V. Hernandez, Toshiki Kuno, Homa Taheri, Robert J. Siegel, Florian Rader, Behnam N. Tehrani, Mohammad Hossein Mandegar, Ehsan Safaee, Pouya Ebrahimi, Kaveh Hosseini

**Affiliations:** ^1^ Imam Khomeini Hospital Complex, Tehran University of Medical Sciences Tehran Iran; ^2^ Tehran Heart Center, Cardiovascular Diseases Research Institute, Tehran University of Medical Sciences Tehran Iran; ^3^ Social Determinants of Health Research Center, Isfahan University of Medical Sciences Isfahan Iran; ^4^ Cardiology Department Modarres Hospital, Shahid Beheshti University of Medical Sciences Tehran Iran; ^5^ Heart Failure Research Center, Cardiovascular Research Institute, Isfahan University of Medical Science Isfahan Iran; ^6^ Faculty of Medicine, Shahid Beheshti University of Medical Sciences Tehran Iran; ^7^ Department of Medicine Division of Nephrology, Stanford University School of Medicine Stanford California USA; ^8^ Cardiology Research Center, Faculty of Medicine, Hamadan University of Medical Sciences Hamadan Iran; ^9^ Health Outcomes, Policy and Evidence Synthesis (HOPES) Group, University of Connecticut School of Pharmacy Storrs Connecticut USA; ^10^ Unidad de Revisiones Sistemáticas y Meta‐análisis (URSIGET), Vicerrectorado de Investigación Universidad San Ignacio de Loyola (USIL) Lima Peru; ^11^ Department of Medicine Montefiore Medical Center New York New York USA; ^12^ Cedars‐Sinai Medical Center Los Angeles California USA; ^13^ Inova Schar Heart and Vascular Falls Church Virginia USA; ^14^ Professor of Cardiovascular Surgery Shariati Hospital, Tehran University of Medical Sciences Tehran Iran; ^15^ Student Research Committee, Faculty of Medicine, Shahed University of Tehran Tehran Iran

**Keywords:** cardiovascular events, meta‐analysis, renal denervation, resistant hypertension, sham‐controlled

## Abstract

**Background:**

Although some guidelines recommend Renal denervation (RDN) as an alternative to anti‐HTN medications, there are concerns about its efficacy and safety. We aimed to evaluate the benefits and harms of RDN in a systematic review and meta‐analysis of sham‐controlled randomized clinical trials (RCT).

**Methods:**

Databases were searched until September 10th, 2024, to identify RCTs evaluating RDN for treating URH versus sham control. The primary outcomes were the change in office and ambulatory 24‐h systolic (SBP) and diastolic blood pressure (DBP). Secondary outcomes were changes in daytime and nighttime SBP and DBP, home BP, number of anti‐HTN drugs, and related complications. Mean differences (MD) and relative risks (RR) described the effects of RDN on BP and complications, respectively, using random effects meta‐analyses. GRADE methodology was used to assess the certainty of evidence (COE).

**Results:**

We found 16 included sham‐controlled RCTs [RDN (*n* = 1594) vs. sham (*n* = 1225)]. RDN significantly reduced office SBP (MD −4.26 mmHg, 95% CI: −5.68 to −2.84), 24 h ambulatory SBP (MD −2.63 mmHg), office DBP (MD −2.15 mmHg), 24‐h ambulatory DBP (MD −1.27 mmHg), and daytime SBP and DBP (MD −3.29 and 2.97 mmHg), compared to the sham. The rate of severe complications was low in both groups (0%–2%). The heterogeneity was high among most indices, and CoE was very low for most outcomes.

**Conclusion:**

RDN significantly reduced several SBP and DBP outcomes versus sham without significantly increasing complications. This makes RDN a potentially effective alternative to medications in URH.

## Graphical abstract

Renal denervation (RDN) holds great promise as a treatment for lowering blood pressure in patients with treatment‐resistant hypertension. It demonstrates significant improvements in both office and ambulatory blood pressure measurements. The procedure is generally safe and effective, with no notable rise in adverse events. Moving forward, future studies should prioritize randomized clinic trials involving diverse populations to improve the applicability of results across different demographics.

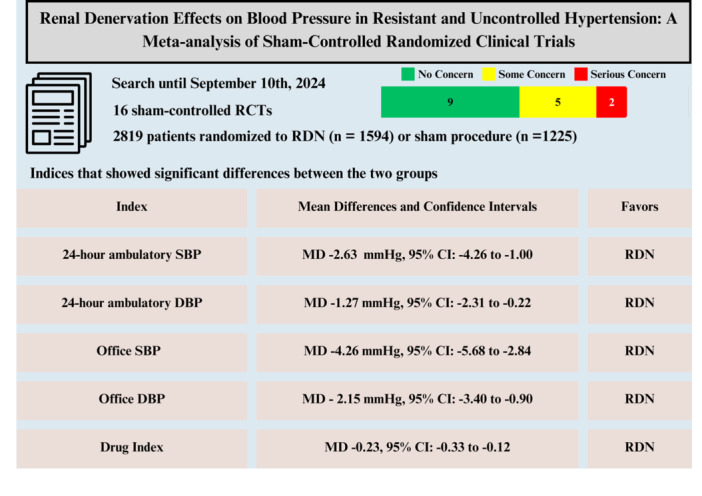

**Abbreviations**
ACE: angiotensin‐converting enzyme
ACM: all‐cause mortality
AKI: acute kidney injury
AMBP: ambulatory blood pressure monitoring
anti‐HTN: antihypertensive
BMI: body mass index
BP: blood pressure
CENTRAL: Cochrane Central Register of Controlled Trials
CI: confidence interval
COE: certainty of evidence
CVA: cerebrovascular accidents
CVD: cardiovascular disease
DBP: diastolic blood pressure
DMII: diabetes mellitus type II
eGFR: estimated glomerular filtration rate
ESC: European Society of Cardiology
ESRD: end‐stage renal disease
FDA: Food and Drug Administration
GFR: glomerular filtration rate
GRADE: Grading of Recommendations Assessment, Development, and Evaluation
HAF: hospitalization for atrial fibrillation
HHF: hospitalization for heart failure
HTN: hypertension
IQR: interquartile range
MACE: major adverse cardiovascular events
MD: mean difference
MI: myocardial infarction
PRISMA: Preferred Reporting Items for Systematic Reviews and Meta‐Analyses
PROSPERO: International Prospective Register of Systematic Reviews
RCT: randomized clinical trial
RDN: renal denervation
REML: restricted maximum likelihood
RoB 2: risk of bias in randomized trials (Version 2)
RR: relative risk
SBP: systolic blood pressure
TRH: treatment‐resistant hypertension
UCH: uncontrolled hypertension
URH: uncontrolled resistant hypertension

## 1 Introduction

Hypertension (HTN) is a main risk factor for cardiovascular disease (CVD), playing a fundamental role in almost one‐fifth of deaths in 2019 [1, 2]. Despite improvement in the pharmacologic treatment of HTN, a substantial percentage of patients have uncontrolled HTN (medicated by two drugs) or drug‐resistant HTN (medicated by three or more drugs, including diuretics). The main causes of these conditions are determined to be nonadherence to medications and insufficient or inappropriate selection of drugs [3]. One of the contributing factors in the development of resistant HTN is sympathetic nerve hyperactivity, especially in obese patients [4]. One of the modern methods of treating HTN is based on deactivating these nerves, a minimally invasive percutaneous procedure called renal denervation (RDN) [5]. Specific types of RDN have been approved by recent guidelines and the Food and Drug Administration (FDA), mentioned as a supplementary or alternative treatment for resistant HTN [6, 7]. However, absolute indications of RDN for treating HTN have not been proposed [8].

Based on the 2023 European Society of Cardiology (ESC) guidelines, RDN can be considered as an option in patients with an estimated glomerular filtration rate (eGFR) > 40 mL/min/1.73 m^2^ who have uncontrolled blood pressure (BP) despite the use of antihypertensive drug (anti‐HTN) combination therapy [9]. RDN is becoming an alternative treatment for patients whose BP cannot be controlled with anti‐HTN medications (resistant HTN). Moreover, previous studies have shown that those with poor medication compliance would benefit from this procedure, considering the decreased or resolved need to take routine drugs after this procedure [10, 11].

Although previous meta‐analyses that investigated the effectiveness and the safety of RDN for the treatment of resistant and uncontrolled and a subgroup analysis of only sham‐controlled trials consistently showed that RDN reduced office and 24‐h SBP [3, 10, 11, 12, 13, 14], there are still unanswered questions. The range of changes differs based on the systolic (SBP) and diastolic blood pressures (DBP), up to 60 mmHg in SBPs and 40 mmHg in DBPs. One of these dilemmas is the superiority of various types of RDN. Three main types of RDN (radiofrequency (RF), ultrasound (US), and alcohol‐mediated denervation) have been evaluated in several RCTs during recent years. Still, the optimal method in various subgroups of patients has yet to be determined [15]. Another reason for conducting this analysis is that, although previous meta‐analyses have been performed, methodological differences between the systematic reviews and the trials included make it challenging to draw generalizable conclusions. In addition, three significant recent trials—TARGET BPI [16], SMART [17], and the Iberis‐HTN Trial by Jiang and colleagues [18]—were not included in two of the most recent meta‐analyses in this field [14, 19]. Hence, we performed a comprehensive meta‐analysis of randomized, sham‐controlled clinical trials investigating the beneficial and harmful effects of RDN on BP across different devices.

## 2 Methods

### 2.1 Protocol and Registration

This systematic review and meta‐analysis adhered to the PRISMA guidelines [20]. The review protocol, with the registration number CRD42024546447, was registered in the International Prospective Register of Systematic Reviews (PROSPERO).

### 2.2 Eligibility Criteria

Studies were considered for inclusion if they satisfied the following criteria: (1) RCTs evaluating any RDN versus a sham procedure in adults aged 18 years or older with resistant HTN; (2) studies providing data SBP and DBP; and (3) studies published in the English language. The exclusion criteria encompassed nonrandomized studies, observational studies, case reports, review articles, editorials, and studies that did not include a sham procedure as a control.

### 2.3 Study Searches

A thorough literature search was performed using PubMed, EMBASE, Scopus, and the Cochrane Central Register of Controlled Trials (CENTRAL) from their inception to July 1st, 2024. The search strategy incorporated terms related to RDN, treatment‐resistant HTN (TRH), resistant and uncontrolled HTN, and randomized controlled trials. The detailed search strategies are available in Supporting Information S2: Table 1. Additionally, reference lists of included articles and relevant reviews were manually screened to identify further studies.

### 2.4 Study Selection and Data Management

Duplicate records were identified and removed using the Rayyan web application, facilitating an efficient and accurate screening process. Rayyan was also used to screen titles and abstracts, allowing independent reviewers (S.H. and E.S.) to classify studies as included, excluded, or marked for further consideration [21]. Full‐text articles of potentially relevant studies were assessed for inclusion. Discrepancies were resolved by consensus or consultation with a third reviewer (R.N.).

### 2.5 Data Extraction

Data extraction was performed using a standardized, predetermined form in Excel. Extracted data included study characteristics (author, year of publication, sample size, follow‐up duration), patient demographics (age, gender, baseline BP, body mass index [BMI]), intervention details (type of RDN, comparator), and outcomes per arm (RDN or sham).

### 2.6 Outcomes and Definitions

Primary outcomes were office and 24‐h ambulatory SBP and DBP. Secondary outcomes include daytime, nighttime, and home SBP and DBP. Moreover, adverse events such as hypertensive and hypotensive emergencies, acute kidney injury (AKI) (sudden rise in creatinine > 50%), hospitalization for heart failure (HHF), hospitalization for atrial fibrillation (HAF), all‐cause mortality (ACM), adverse event rate, severe adverse event rate, severe cerebrovascular adverse event rate, new end‐stage renal disease (ESRD), embolic events accompanied by end‐organ damage, hospitalization for major adverse cardiovascular events (MACE) (cerebrovascular accidents (CVA), myocardial infarction (MI)), new renal artery stenosis > 70%, major vascular complication (renal artery perforation or dissection). Moreover, the changes in the number of anti‐HTN drugs and anti‐HTN drug index were measured. Definitions of resistant HTN and clinical outcomes followed standard criteria described in the included studies.

– **Resistant Hypertension:** It is defined as above‐goal elevated BP in patients despite the concurrent use of ≥ 3 anti‐HTN medications from various classes, commonly including a long‐acting calcium channel blocker (CCB), a blocker of the renin–angiotensin system (angiotensin‐converting enzyme inhibitor or angiotensin receptor blocker), and a diuretic [22].
– **Uncontrolled Hypertension:** A BP above 140/90, which might be caused secondary to poor adherence or an inadequate treatment regimen, as well as those with true treatment resistance [22].
– **Drug Index:** It is constructed as Class *N* (number of classes of antihypertensive drugs) × (sum of doses) [23].

### 2.7 Risk of Bias Assessment

Two independent authors (H.T. and M.T.) evaluated the risk of bias in individual RCTs using the Cochrane Collaboration tool for assessing the risk of bias in randomized trials (RoB 2) [24]. Discrepancies between the reviewers were resolved by consensus. RoB 2 assessments addressed five domains, including the randomization process, deviations from intended interventions, missing outcome data, measurement of outcomes, and selection of the reported results. Each domain and each RCT was then categorized as having a low, high, or unclear risk of bias [24].

### 2.8 Statistical Analyses

Statistical analyses were conducted with the R software [25] (R for Windows, version 4.1.3, Vienna, Austria) and R Studio version 1.1.463 (Posit PBC, Boston, MA, USA), utilizing the tidyverse [26] and meta [27] packages.

Pooled RDN effects versus medical treatment on BP outcomes were expressed as mean differences (MD) with 95% confidence intervals (CI); binary outcomes were expressed as relative risks (RR) with 95% CI. The between‐study variance was described with the *τ* ^2^ estimate. Random‐effect models were used to account for variability across studies. When studies reported medians and interquartile ranges (IQR), the method developed by Lou and colleagues [28] and Wan and colleagues [29] was used to calculate the mean and SD. If the SD of the mean change was not reported, it was calculated using the formula [30]:

$$\mathrm{SD}\;\mathrm{change}=\sqrt{\left({\mathrm{SD}}_{\text{baseline}}^{2}+{\mathrm{SD}}_{\text{final}}^{2}\right)-(2\times 0.6\times {\mathrm{SD}}_{\text{baseline}}\times {\mathrm{SD}}_{\text{final}})}.$$

Meta‐analyses were performed using a random‐effects model with restricted maximum likelihood (REML) estimation. Heterogeneity among studies was assessed using Cochrane's *Q* statistic and *I* ^2^ statistic [31], with *I* ^2^ values classified as low (< 25%), moderate (25%–50%), or high (> 50%) heterogeneity. Visual and statistical assessments for publication bias were conducted using funnel plots and Begg's and Egger's tests [32].

Subgroup analyses by device type (alcohol, RF, and US) and medication status (on vs. off) were conducted. A *p* for interaction < 0.10 meant a significant subgroup effect for an outcome. Sensitivity analyses were performed using three methods: (1) by using a fixed‐effect model, (2) by using a leave‐one‐out sensitivity analysis, and (3) by excluding the studies that had < 100 participants in each arm. Meta‐regression analysis was conducted for the outcomes of 24‐h SBP and DBP with variables of age, BMI, glomerular filtration rate (GFR), male proportion, baseline mean SBP, and follow‐up duration. Certainty of evidence and strength of recommendations were performed using the Grading of Recommendations Assessment, Development, and Evaluation (GRADE) Guidelines. A summary of COE was performed based on the evaluation of the pooled studies' number for each index and their design, indirectness, imprecision, and the number of included patients.

## 3 Results

### 3.1 Study Selection

**Figure 1:**
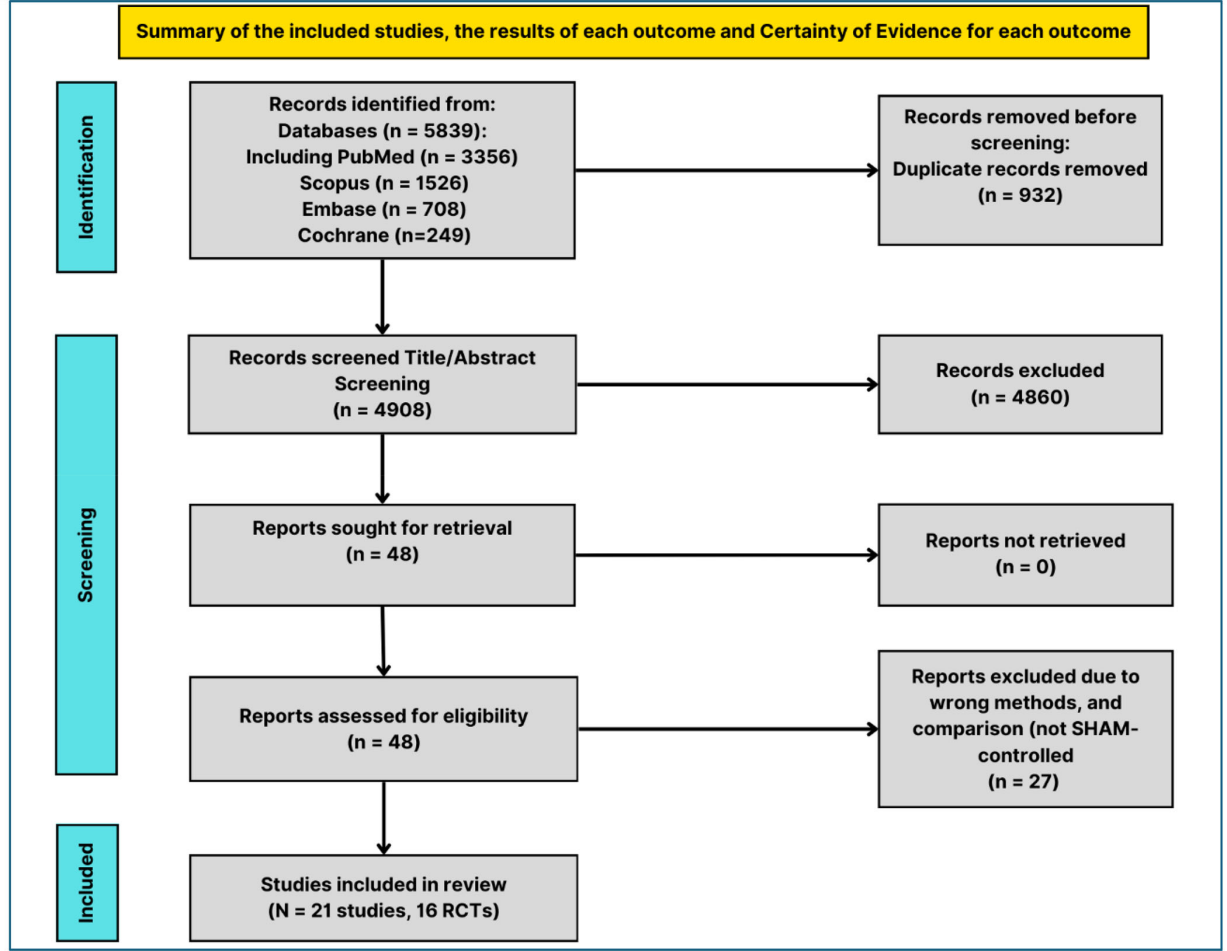
PRISMA flow diagram of included studies.

A comprehensive literature search identified a total of 5839 records from various databases, including PubMed (*n* = 3356), Scopus (*n* = 1526), Embase (*n* = 708), and Cochrane CENTRAL (*n* = 249). After excluding 932 duplicates, 4907 unique records were screened based on titles and abstracts. During this initial screening, 4860 records were excluded for not meeting the inclusion criteria. Consequently, 47 full‐text articles were retrieved for a detailed evaluation. After further assessment, 25 articles were excluded due to not aligning with the required methods, population, or intervention/comparison criteria. Ultimately, 21 reports from 16 randomized controlled trials (RCTs) were included in the systematic review and meta‐analysis (Figure 1) [16, 17, 18, 33, 34, 35, 36, 37, 38, 39, 40, 41, 42, 43, 44, 45, 46, 47, 48, 49, 50].

The selected studies comprised a total of 2819 participants (RDN: 1594 and sham procedure: 1225), with sample sizes ranging from 51 to 515 participants per study. Follow‐up periods were varied, with the majority ranging from 2 to 12 months, although some extended up to 36 months. Participants' demographics showed a broad range of ages, with the mean age of participants spanning from 44.5 to 65.5 years. The mean BMI across studies ranged from 28.2 to 34.2 kg/m^2^. The proportion of male participants also varied; some studies had as few as 18% male participants, while others had up to 95.5%. The included studies also reported diverse distributions of diabetes mellitus (DMII) prevalence and the number of anti‐HTN medications taken by participants (Table 1).

**Table 1:** Primary characteristics of included trials.

Trials, author/acronym, year (reference)	NCT	Country	Device	Energy source	Scape criteria	FU (M)	Number	
							RD	Sham
1. Symplicity HTN‐3, Bhatt, 2014 [33]	NCT01418261	USA	Symplicity renal‐denervation catheter (Medtronic)	RF	No escape criteria are defined in this trial	6	364	171
2. Symplicity FLEX, Desch, 2015 [34]	NCT01656096	Germany	Symplicity Flex catheter (Medtronic)	RF‐RDN	No escape criteria are defined in this trial	6	35	36
3. ReSET, Mathiassen, 2016 [35]	No NCT	Denmark	Simplicity renal denervation catheter (Medtronic)	RF‐RDN	No escape criteria are defined in this trial	6	36	33
4. Wave IV, Schmieder, 2018 [36]	No NCT	Czech Republic, Germany, New Zealand, Poland, UK	Kona Surround Sound system	US‐RDN	No escape criteria are defined in this trial	6	42	39
5. RADIANCE‐HTN SOLO, Azizi, 2018 [37]	NCT02649426	USA, France, Germany, the Netherlands, Belgium, UK	Paradise RDN system (ReCor Medical)	US‐RDN	OBP > 180/110 mmHg or HBP > 170/105 mmHg	2	74	72
5. RADIANCE‐HTN SOLO, Azizi, 2019 [38]						6		
5. RADIANCE‐HTN SOLO, Azizi, 2019 [39]						12		
6. SPYRAL HTN‐OFF MED Pivotal, Bohm, 2020 [50]	NCT02439749	Australia, Austria, Canada, Germany, Greece, Ireland, Japan, UK, USA	Symplicity Spyral multielectrode catheter (Medtronic, Mountain View, CA) or Symplicity G3 radiofrequency generator (Medtronic)	RF‐RDN	OBP > 180/110 mmHg or HBP > 170/105 mmHg	3	166	165
7. REINFORCE, Weber, 2020 [40]	NCT02392351	USA	Vessix Renal Denervation system	RF‐RDN	Office SBP > 180 mmHg at two consecutive visits within 3 days	2	34	17
8. RADIANCE‐HTN TRIO, Azizi, 2021 [41]	NCT02649426	USA, France, Germany, the Netherlands, Belgium, Poland, and UK	Paradise RDN system (ReCor Medical)	US‐RDN	OBP > 180/110 mmHg or HBP > 170/105 mmHg	2	69	67
8. RADIANCE‐HTN TRIO, Azizi, 2022 [42]						6	69	67
9. REQUIRE, Kario, 2021 [43]	NCT02918305	Japan and S. Korea	Paradise RDN system (ReCor Medical)	RF‐RDN	No escape criteria are defined in this trial	3	72	71
10. Heradien 2022 [44]	NCT01990911	South Africa	Symplicity Flex and Spyral	RF		6	42	38
11. TARGET BP OFF‐MED, Pathak, 2023 [45]	NCT03503773	US, several European countries	Peregrine System Infusion Catheter (Ablative Solutions Inc)	Alcohol‐RDN	No escape criteria are defined in this trial	2	50	56
12. RADIANCE II, Azizi, 2023 [46]	NCT03614260	Belgium, France, Germany, the Netherlands, Poland, and UK	Paradise system (ReCor Medical, Inch Palo Alto, CA)	US‐RDN	OBP > 180/110 mmHg or HBP > 170/105 mmHg	2	150	74
13. SPYRAL ON MED (expansion) Kandazari 2023 [49]	NCT02439775	USA, Germany, Japan, UK, Australia, Austria, and Greece	Symplicity Spyral multielectrode catheter (Medtronic) or Symplicity G3 radio‐frequency generator (Medtronic)	RF‐RDN	OBP > 180/110 mmHg or HBP > 170/105 mmHg	6	206	131
13. SPYRAL ON MED, Mahfoud (expansion) 2023 [47]						24	34	17
13. SPYRAL ON MED (expansion) Kario, 2022 [48]						36	36	42
14. TARGET BPI, Kandazari, 2024 [16]	NCT02910414	USA	Peregrine	Alcohol		6	145	146
15. SMART, Wang, 2024 [17]	NCT02761811	China	Catheter	RF		6	109	110
16. Iberis‐HTN Trial, Jiang, 2024 [18]	NCT02901704	China	Multielectrode, unipolar Iberis RDN catheter and generator system (AngioCare, Shanghai, China)	low‐level RF	Symptomatic hypertension or SBP ≥ 180 mmHg or symptomatic hypotension or systolic BP	6	107	110

Abbreviations: FU (M), follow‐up (months); HBP, home blood pressure; NCT, National Clinical Trial number; OBP, office blood pressure; RD, renal denervation; RF, radiofrequency; RF‐RDN, radiofrequency renal denervation; SBP, systolic blood pressure; US‐RDN, ultrasound renal denervation.

### 3.2 Quality Assessment of Included Trials

The quality assessment of the included articles was conducted using the risk of bias in randomized trials (RoB 2) [24]. Overall, five studies' evaluations revealed a high risk of bias, while two others were categorized as having some concerns about bias. Nine others showed a low risk of bias (Supporting Information S1: Figure 1).

### 3.3 Effects of RDN on Primary Outcomes

#### 3.3.1 24‐h Ambulatory Blood Pressures

**Figure 2:**
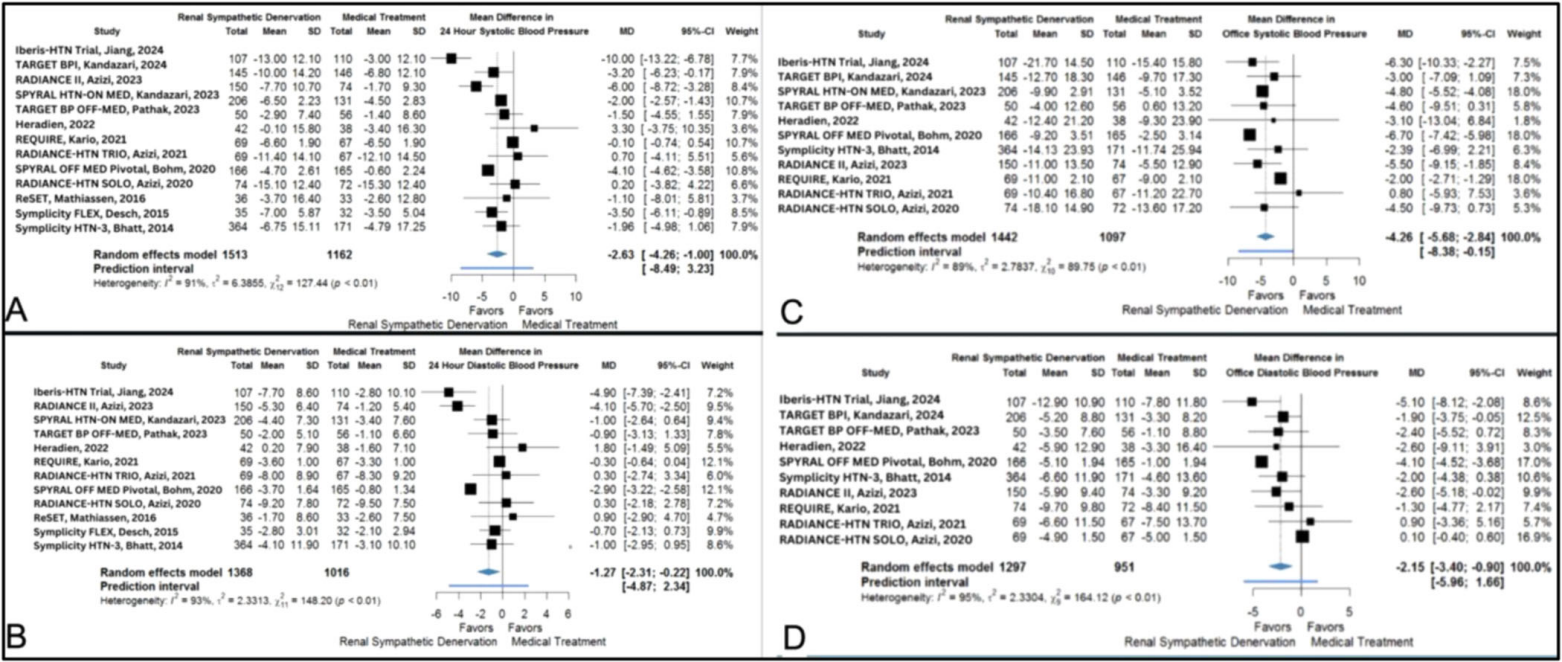
Effect of RDN versus medical treatment on (A) 24‐h systolic blood pressure, (B) 24‐h diastolic blood pressure, (C) office systolic blood pressure, and (D) office diastolic blood pressure.

RDN significantly reduced 24‐h SBP (MD −2.30 mmHg, 95% CI: −3.53 to −1.07) (Figure 2A) and DBP (MD −1.21 mmHg, 95% CI: −2.18 to −0.23) compared to medical treatment (Figure 2B). Of the 11 trials pooled in the 24‐h SBP calculation, RADIANCE II (2023) had the most prominent difference between the two groups (MD −6.00 mmHg, 95% CI: −8.72 to −3.28), while four other studies revealed a statistically considerable difference between two categories, but the MD was lower. Ten studies were analyzed in the pooled data of 24‐h DBP; seven trials showed a more prominent decrease in the MD, and three showed an insignificantly higher reduction in this index in the control group. The heterogeneity among studies included in the pooled analyses of these two indices was high, showing *I* ^2^ of 90% for 24‐h SBP and *I* ^2^ of 93% for 24‐h DBP.

#### 3.3.2 Office Blood Pressures

A similar pattern could be seen in the results of the office BP measurements, where RSD significantly reduced office SBP (MD −4.11 mmHg, 95% CI: −5.64 to −2.57) (Figure 2C) and office DBP (MD −1.85 mmHg, 95% CI: −3.15 to −0.55) (Figure 2D) in comparison with medical treatment. However, like 24‐h BPs, the included studies in both pooled analyses showed a high heterogeneity. Among nine studies included in the pooled analysis of office SBP, SPYRAL HTN‐OFF MED Pivotal (2020) demonstrated the most substantial reduction in the SBP (MD −6.70 mmHg, 95% CI: −7.42 to −5.98), while RADIANCE‐HTN TRIO (2022) was the only study which revealed the increased mean of SBP in the RDN group compared to the control group. However, the difference was not significant. Eight studies were included in the pooled analysis of office DBP; six showed a more substantial decrease in DBP, but only three showed significant differences. The measured *I* ^2^ for office SBP and DBP were 91% and 96%, respectively.

#### 3.3.3 Effects of RDN on Secondary Outcomes

**Figure 3:**
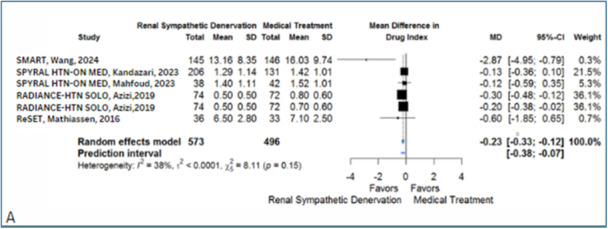
Effect of renal denervation versus medical treatment on (A) the drug index.

Among the secondary outcomes, the MD between the RDN and control group was insignificant in the pooled analysis of the night‐time BPs (Supporting Information S1: Figure 2), home BPs (Supporting Information S1: Figure 3), and daytime DBP RDN (Supporting Information S1: Figure 4B). However, this intervention significantly reduced daytime SBP versus medical treatment (MD −3.29 mmHg, 95% CI: −5.43 to −1.15) (Supporting Information S1: Figure 4A). The heterogeneity between studies was significant among the nine included trials in this study (*I* ^2^ = 80%) (Supporting Information S2: Table 2).

Although there was no significant difference in the reduction of the number of anti‐HTN medications between RDN and medical treatment (MD −0.08, 95% CI: −0.25 to 0.10) (Supporting Information S1: Figure 5), the drug index pooled analysis showed a significantly lower MD in the RDN group (MD = −0.23, 95% CI: −0.33 to −0.12) (Figure 3 and Supporting Information S2: Tables 3). Six studies included in this index revealed insignificant heterogeneity (*I* ^2^ = 38%).

The pooled analysis of the adverse outcomes, including embolic event which caused end‐organ damage, major access site complications, procedure‐related pain > 2 days, severe cerebrovascular adverse event rate, AKI, ACM, major vascular complications, HTN emergency, hypotension emergency, CVA, MI, creatinine rise, renal artery stenosis, percutaneous coronary intervention, HHF and HAF showed an insignificant rate (Supporting Information S2: Table 4) and also a negligible difference between risk ratios (RR) of RDN and medication group. The incidence of HTN and hypotension emergencies also showed insignificant rates and insignificant differences (Supporting Information S1: Figure 6).

### 3.4 Sensitivity and Subgroup Analyses

#### 3.4.1 The 24‐h Ambulatory Mean of Systolic and Diastolic Blood Pressures

Fixed‐effect and random‐effect (leave‐one‐out) methods were employed for sensitivity analyses. The results from the pooled data for the random‐effect (leave‐one‐out) approach demonstrated a significant difference between groups in 24‐h SBP, with considerable heterogeneity (MD: −2.42 mmHg, 95% CI: −2.74 to −2.11, *p* < 0.01) (Supporting Information S1: Figure 7). Using a random‐effects model, the MD in larger studies (sample size > 100) was even larger (MD of −3.41 mmHg, 95% CI: −5.10 to −1.72) (Supporting Information S1: Figure 8). The subgroup analysis of the 24‐h SBP outcomes based on device type and medication status revealed significant differences between the intervention and control groups except for the US‐based device group (Supporting Information S1: Figures 9 and 10).

The fixed effect model showed a significant reduction of 24 h DBP (MD; 1.63 mmHg (95% CI: −1.84 to −1.41)) (Supporting Information S1: Figure 11A), and the sensitivity analyses using the leave‐one‐out method resulted in a pooled MD of −1.27 mmHg (95% CI: −2.31 to −0.22, *p* = 0.02) (Supporting Information S1: Figure 11B). In larger studies, the random‐effects model yielded a MD of −2.36 mmHg (95% CI: −3.75 to −0.97) (Supporting Information S1: Figure 12). The subgroup analysis of the 24‐h SDP outcomes based on the device type and medication status revealed significant differences between intervention and control groups except for US and alcohol‐based devices groups (Supporting Information S1: Figures 13 and 14).

#### 3.4.2 Office Systolic and Diastolic Blood Pressure

The fixed effect model showed a significant reduction of office SBP (MD: −4.45 mmHg (95% CI: −4.85 to −4.05)) (Supporting Information S1: Figure 15A), and the sensitivity analyses using the leave‐one‐out method resulted in a pooled MD of −4.26 mmHg (95% CI: −5.68 to −2.84, *p* < 0.01) (Supporting Information S1: Figure 15B). In larger studies, the random‐effects model yielded a MD of −5.44 mmHg (95% CI: −6.93 to −3.95) (Supporting Information S1: Figure 16). The subgroup analysis by the type of device showed significant differences between the intervention and control groups in RF and US‐based devices (Supporting Information S1: Figure 17).

The fixed effect model showed a reduction in office DBP by 2.38 mmHg (95% CI: −2.68 to −2.07), with substantial heterogeneity (*I* ^2^ = 96%) (Supporting Information S1: Figure 18A). Sensitivity analyses using the leave‐one‐out method resulted in a pooled MD of −2.15 mmHg (95% CI: −3.40 to −0.90, *p* < 0.01) with substantial heterogeneity (*I* ^2^ = 96%) (Supporting Information S1: Figure 18B). Additionally, a random‐effects model including large studies and a separate supplementary analysis with a reduced data set confirmed a similar significant reduction in office DBP, with a pooled MD of −2.95 mmHg (95% CI: −4.28 to −1.63) and moderate heterogeneity (*I* ^2^ = 66%) (Supporting Information S1: Figure 19). The subgroup analysis by the type of device showed significant differences between the intervention and control groups in RF‐based methods and insignificant differences between groups in US‐based devices (Supporting Information S1: Figure 20).

#### 3.4.3 Home Systolic and Diastolic Blood Pressure

The fixed effect model also showed a reduction of home SBP by 1.78 mmHg (95% CI: −2.38 to −1.19), with low heterogeneity (*I* ^2^ = 0%) (Supporting Information S1: Figure 21A). Sensitivity analyses using the leave‐one‐out method resulted in a pooled MD of −1.78 mmHg (95% CI: −2.38 to −1.19, *p* < 0.01) with no heterogeneity (*I* ^2^ = 0%) (Supporting Information S1: Figure 21B). The fixed effect model also showed an increase of home DBP by 0.07 mmHg (95% CI: −0.29 to 0.42), with low heterogeneity (*I* ^2^ = 0%) (Supporting Information S1: Figure 22A). Sensitivity analyses using the leave‐one‐out method resulted in a pooled MD of 0.07 mmHg (95% CI: −0.29 to 0.42, *p* = 0.07) with no heterogeneity (*I* ^2^ = 0%) (Supporting Information S1: Figure 22B).

#### 3.4.4 Nighttime Systolic and Diastolic Blood Pressure

The fixed effect model showed a reduction in nighttime SBP by 2.01 mmHg (95% CI: −2.51 to −1.51), with substantial heterogeneity (*I* ^2^ = 90%) (Supporting Information S1: Figure 23A). Sensitivity analyses using the leave‐one‐out method resulted in a pooled MD of −2.14 mmHg (95% CI: −4.58 to 0.30, *p* = 0.19) with substantial heterogeneity (*I* ^2^ = 90%) (Supporting Information S1: Figure 23B). The subgroup analysis by the type of device showed insignificant differences between the intervention and control groups in both RF‐ and US‐based devices (Supporting Information S1: Figure 24). The fixed effect model showed an increase in nighttime DBP by 0.28 mmHg (95% CI: −0.12 to 0.68), with substantial heterogeneity (*I* ^2^ = 86%) (Supporting Information S1: Figure 25A). Sensitivity analyses using the leave‐one‐out method resulted in a pooled MD of −0.81 mmHg (95% CI: −2.85 to 1.22, *p* = 0.43) with substantial heterogeneity (*I* ^2^ = 86%) (Supporting Information S1: Figure 25B). Interestingly, the subgroup analysis by the type of device showed a significantly lower MD of DBP in the intervention group in RF‐based methods, while the differences between the intervention and control groups in the US‐based devices were insignificant (Supporting Information S1: Figure 26).

#### 3.4.5 Daytime Systolic and Diastolic Blood Pressure

The fixed effect model reduced daytime SBP by 1.70 mmHg (95% CI: −2.13 to −1.26) (Supporting Information S1: Figure 27A). Sensitivity analyses using the leave‐one‐out method resulted in a pooled MD of −3.29 mmHg (95% CI: −5.43 to −1.15, *p* < 0.01) with substantial heterogeneity (*I* ^2^ = 80%) (Supporting Information S1: Figure 27B). The subgroup analysis by the type of device showed significant differences between the intervention and control groups in the RF‐ and the insignificant differences between the two groups in US‐based devices (Supporting Information S1: Figure 28). The fixed effect model showed a reduction in daytime DBP by 7.92 mmHg (95% CI: −8.24 to −7.59), with substantial heterogeneity (*I* ^2^ = 97%) (Supporting Information S1: Figure 29A). Sensitivity analyses using the leave‐one‐out method resulted in a pooled MD of −2.97 mmHg (95% CI: −5.64 to −0.30, *p* = 0.1) with substantial heterogeneity (*I* ^2^ = 97%) (Supporting Information S1: Figure 29B). The subgroup analysis by the type of device showed significant differences between the intervention and control groups in the RF‐based and insignificant differences between the intervention and control groups in the US‐based devices (Supporting Information S1: Figure 30).

#### 3.4.6 Antihypertensive Medications Number and Drug Index

The fixed effect model showed a reduction of the number of anti‐HTN medications by −0.06 (95% CI: −0.16 to 0.03) with substantial heterogeneity (*I* ^2^ = 70%) (Supporting Information S1: Figure 31A). Sensitivity analyses further support these results, with a random effects model showing a MD of −0.08 (95% CI: −0.25 to 0.10, P:0.38) and similar heterogeneity (*I* ^2^ = 73%) (Supporting Information S1: Figure 31B). Additionally, a random‐effects model, including large studies, showed a MD of −0.03 (95% CI: −0.38 to 0.32), with substantial heterogeneity (*I* ^2^ = 89%, *p* < 0.01) (Supporting Information S1: Figure 32).

The fixed effect model showed a significant reduction in the drug index in the RDN group compared to the control group by an MD of −0.23 (95% CI: −0.33 to −0.12) with insignificant heterogeneity (*I* ^2^ = 38%) (Supporting Information S1: Figure 33A). The random effects model, using the leave‐one‐out method, showed a statistically significant difference between the intervention and control group with an MD of −0.23 (95% CI: −0.33 to −0.12, *p* < 0.01) and similar heterogeneity (*I* ^2^ = 38%) (Supporting Information S1: Figure 33B). The comparison between random effect and fixed‐effect analysis revealed differences in some indices. This comparison highlights the variability and consistency in effect size estimates between the two meta‐analytical approaches, indicating that the nighttime results are statistically significant. All effects are MD between RDN and sham in mmHg (Supporting Information S2: Table 5).

Overall, only RF‐based devices demonstrated significant differences in 24‐h DBP, office DBP, and day SBP subgroup analysis (Supporting Information S1: Figures 13, 20, and 28). The subgroup analysis based on the on‐ and off‐med subgroups in 24‐h SBP and DBP showed that although the difference between both subgroups was significant, pooled MD of off‐med subgroup were more prominent than on‐med patients in both mentioned indices (Supporting Information S1: Figures 9 and 14).

#### 3.4.7 Meta‐Regression and Publication Bias

The 24‐h SBP and DBP meta‐regression analyses showed nonsignificant trends for age, BMI, and the proportion of male patients, with wide CI (Supporting Information S1: Figure 34A–F). Significant associations were found for GFR, baseline mean SBP, and follow‐up time, with narrower CI and consistent data patterns (Supporting Information S1: Figure 34G–L).

The sham‐controlled design of some trials may have introduced bias, as it is unclear whether participants in the sham group were effectively blinded, potentially influencing the outcomes. However, a *p* value of 0.18 (Supporting Information S1: Figure 35) indicates no statistically significant evidence of asymmetry in evaluating the number of anti‐HTN medications. Therefore, publication bias is unlikely. Moreover, a time‐trend analysis of three trials with 2–12‐month follow‐ups showed that the MD of 24‐h AMBP had an overall increasing trend in systolic and diastolic indexes (Supporting Information S1: Figure 36).

### 3.5 Certainty of Evidence (COE)

Table 2 presents the GRADE summary of the findings table with COE assessments. Moreover, two independent assessors (P.E. and A.E). were invited to assess the certainty of evidence. Disagreements were referred to a third senior author (K.H.), who asked three researchers and a professional coauthor to the GRADE PRO platform to revise the process. We chose the seven most important indicators of the certainty of evidence. However, considering Cochrane's standards, 24‐h office SBP and DBO and changes in the number of anti‐HTN were categorized as very low certainty, and the drug index was classified as low certainty of evidence. The main causes of downgrading of COE were as follows: (1) studies that carried large weight for the overall effect estimate rated high risk of bias due to missed outcomes. (2) The rate of heterogeneity is very high. (3) Many studies have shown bias due to a high rate of missed outcomes and/or lack of appropriate concealment. (4) A lower number of studies report the index.

**Table 2:** Certainty of evidence based on GRADE criteria has been determined for indices.

Certainty assessment							No. of patients		Effect	Certainty	Importance
No. of studies	Study design	Risk of bias	Inconsistency	Indirectness	Imprecision	Other considerations	RDN	SHAM‐controlled	Absolute (95% CI)		
24‐h systolic blood pressure											
13	Randomized trials	Serious^a^	Very serious^b^	Not serious	Not serious	None	1513	1162	MD 2.63 mmHg lower (4.66 lower to 1 lower)	⊕◯◯◯ Very low^a^, ^b^	Critical
24‐h diastolic blood pressure											
12	Randomized trials	Serious	Very serious^b^	Not serious	Not serious	None	1368	1016	MD 1.27 mmHg lower (2.31 lower to 0.22 lower)	⊕◯◯◯ Very low^b^, ^c^	Critical
Office systolic blood pressure											
11	Randomized trials	Serious^a^	Very serious^b^	Not serious	Not serious	None	1442	1097	MD 4.26 mmHg lower (5.68 lower to 2.54 lower)	⊕◯◯◯ Very low^a^, ^b^	Critical
Office diastolic blood pressure											
10	Randomized trials	Serious^c^	Very serious^b^	Not serious	Not serious	None	1297	951	MD 2.15 mmHg lower (3.4 lower to 0.9 lower)	⊕◯◯◯ Very low^b^, ^c^	Critical
Daytime systolic blood pressure											
9	Randomized trials	Serious	Very serious^b^	Not serious	Not serious	None	891	732	MD 3.29 mmHg lower (5.43 lower to 1.15 lower)	⊕◯◯◯ Very low^b^, ^d^	Critical
Daytime diastolic blood pressure											
7	Randomized trials	Serious^d^	Very serious^b^	Not serious	Not serious	None	433	345	MD 2.97 mmHg lower (5.54 lower to 0.3 lower)	⊕◯◯◯ Very low b, d	Critical
Night systolic blood pressure											
9	Randomized trials	Serious^d^	Very serious^b^	Not serious	Not serious	None	891	732	MD 2.14 mmHg lower (4.58 lower to 30 higher)	⊕◯◯◯ Very low^b^, ^d^	Important
Night diastolic blood pressure											
7	Randomized trials	Serious^c^, ^d^	Serious^b^	Not serious	Not serious	None	433	345	MD 0.81 mmHg lower (2.85 lower to 0.22 higher)	⊕⊕◯◯ Low^b^, ^c^, ^d^	Important
Home diastolic blood pressure											
4	Randomized trials	Not serious	Not serious	Not serious	Not serious	None	540	452	MD 0.17 mmHg lower (2.14 lower to 1.8 higher)	⊕⊕⊕⊕ High	Important
Home systolic blood pressure											
3	Randomized trials	Not serious	Not serious	Not serious	not serious	None	639	476	MD 1.05 mmHg lower (3.34 lower to 1.24 higher)	⊕⊕⊕⊕ High	Important
Drug index											
6	Randomized trials	Serious^d^	Not serious	Not serious	Serious^e^	None	573	496	MD 0.23 mmHg lower (0.33 lower to 0.12 lower)	⊕⊕◯◯ Low^d^, ^e^	Critical
Number of antihypertensive drugs											
10	Randomized trials	Serious^a^	Serious^b^	Not serious	Serious^e^	None	1297	935	MD 0.08 mmHg lower (0.25 lower to 0.1 lower)	⊕◯◯◯ Very low^a^, ^b^, ^e^	Critical

Abbreviations: CI, confidence interval; MD, mean difference.

^a^ Studies that carried large weight for the overall effect estimate rated a high risk of bias due to missed outcomes.

^b^ The rate of heterogeneity is very high (*I* ^2^ > 75).

^c^ Many studies have shown bias due to a high rate of missed outcomes and/or lack of appropriate concealment.

^d^ It is reported by a lower number of studies.

^e^ The calculation of this index is based on subjective measures and patients' reports.

## 4 Discussion

This meta‐analysis represents the largest review of randomized, sham‐controlled trials evaluating RDN for TRH, encompassing 21 articles from 16 trials with 2819 patients. Our findings provide updated insights into the effectiveness of RDN in managing HTN. The analysis reveals that RDN significantly reduces office and 24‐h BP, including systolic and diastolic measures. However, the effect on home and nocturnal BP measurements was inconsistent. RDN significantly lowered 24‐h systolic BP and diastolic BP compared to medical treatment. These results indicate a substantial effect on BP reduction, although the heterogeneity among studies was high. Significant reductions were observed in office BP measurements, though again with high heterogeneity. For secondary outcomes, RDN showed a significant decrease in daytime systolic BP but no significant effect on nighttime BP or home BP measurements. The reduction in the number of anti‐HTN medications was not statistically significant, though the drug index analysis revealed a significant decrease. The adverse events associated with RDN were not significantly different from those in the control group, suggesting that RDN is a safe procedure. However, the high heterogeneity among studies and variability in BP measurement methods indicate the need for further research to refine the use of RDN in clinical practice.

The effectiveness of anti‐HTN treatments in reducing major cardiovascular events is often linked to their impact on ABPM. For example, Dolan and colleagues (2007) reported that reductions in ABPM SBP and nighttime DBP are associated with decreased cardiovascular mortality [51]. Similarly, Mancia and colleagues found that increased erratic variability in DBP was significantly associated with higher cardiovascular death risk [52]. Another trial demonstrated that a ≥ 5 mmHg decline in ABPM was linked to a significant reduction in MACE [53]. Our meta‐analysis supports these findings, showing significant decreases in ABPM with RDN. This aligns with Ogoyama and colleagues's recent meta‐analysis, which found RDN effective in reducing 24‐h SBP [10]. However, our results diverge from studies regarding the effect on daytime BP. While Ogoyama and colleagues [10] reported significant short‐term effects of RDN on daytime SBP, other studies did not find substantial differences [11, 49]. Our subgroup analysis indicates that radiofrequency‐based RDN showed significant differences in daytime BP, while US‐based treatments did not, highlighting the need to consider device type when evaluating RDN efficacy.

Beyond office BP measurements, nocturnal and home BP reductions in TRH (≥ 3 anti‐HTN medications, including at least one diuretic) are associated with fewer cardiovascular events and deaths [54, 55, 56, 57, 58]. For example, Kario and colleagues (2021) found that a nocturnal BP ≥ 120/70 mmHg is linked to a higher risk of heart failure [43]. ESH 2023 guidelines recommend monitoring BP variability, including home BP measurements, to assess treatment efficacy [9]. Our study found RDN significantly affects daytime SBP but not nighttime BP or home BP measurements. In addition, there is conflicting data regarding RDN's effect on daytime BP. Ogoyama and colleagues (2024) reported positive short‐term effects of RDN on daytime SBP [10], while another review, including nine RCTs, did not find significant differences between RDN and control groups [30]. Our study aligns with Ogoyama and colleagues's findings, showing a significant difference in daytime SBP but not DBP. Subgroup analysis indicated that radiofrequency‐based RDN was more effective in reducing daytime SBP than US‐based treatments, highlighting the impact of device type on treatment efficacy.

Our analysis indicates that RDN is effective regardless of concurrent anti‐HTN medication use, consistent with literature suggesting that RDN's benefits are not diminished by medication use [37, 59]. Therefore, it can be understood that taking anti‐HTN medications as a boosting strategy to enhance the decline of BP in patients who have undergone RDN has no negative impact on the therapeutic effect of this intervention [10]. Nonadherence to anti‐HTN medications, common in patients with resistant HTN, can worsen BP fluctuations and cardiovascular outcomes [60]. RDN offers a stable BP reduction, potentially providing a clinical advantage over medication alone by mitigating adherence issues. The dependency on anti‐HTN medications affects their concentration in the blood; the lower level might. Previous studies have suggested that RDN may reduce the average number of anti‐HTN medications and improve quality of life [17, 49, 59, 61]. However, our analysis did not find significant differences in medication numbers between RDN and control groups. More controlled studies are needed to clarify the impact of RDN on medication use. RDN's advantage in providing a stable BP reduction may improve quality of life by reducing medication side effects and concerns about adherence. RDN's long‐term impact on office BP and reduction in MACE incidence is promising [53, 62]. While many RCTs have evaluated MACE composites, direct assessments of RDN's impact on MACE incidence are limited. The Simplicity Registry reported a 35% reduction in MACE incidence with RDN [41]. Our study supports the effectiveness of RDN in reducing office BPs and suggests its potential for improving quality of life and reducing MACE.

### 4.1 Limitations and Strengths

Several limitations should be considered when interpreting the findings of this meta‐analysis. First, the high heterogeneity observed across studies, particularly in 24‐h and office BP measurements, limits the generalization of results. The variation in study design, RDN devices, and patient populations may contribute to this heterogeneity. Second, not all studies consistently reported on secondary BP outcomes such as home, daytime, and nocturnal BP, leading to incomplete data for these parameters. Third, the trials included in this analysis had relatively short follow‐up periods, limiting insights into the long‐term efficacy and safety of RDN.

Additionally, the impact of anti‐HTN medications was not fully addressed, as variations in medication adherence among participants could have confounded the results. The strength of our meta‐analysis lies in its emphasis on sham‐controlled trials, which bolsters the reliability of our conclusions. By incorporating recent studies and applying the GRADE approach to evaluate the quality of evidence, we provide a strong foundation for informed clinical decision‐making.

### 4.2 Conclusion and Recommendations

In conclusion, RDN is a promising treatment for reducing BP in patients with TRH. It shows significant benefits in office and ambulatory BP measurements. The procedure appears safe and effective, with no substantial increase in adverse events. Further studies should prioritize conducting randomized controlled trials with diverse populations to enhance the generalizability of findings across various demographics. Additionally, research should focus on evaluating variations in study designs, particularly those with narrower CI favoring the intervention, to understand factors influencing outcomes. Finally, performing cost‐effectiveness analyses will provide critical insights to guide clinical decision‐making and resource allocation better. Therefore, future studies should focus on standardizing the measurement of BP outcomes, extending follow‐up durations, and rigorously addressing medication adherence to better define the role of RDN in clinical practice.

## Author Contributions

Hamidreza Soleimani, Babak Sattartabar, Bahar Parastooei, Reza Eshraghi, Ehsan Safaee, Roozbeh Nazari, Soroush Najdaghi, Pouya Ebrahimi, Kaveh Hosseini, and Sara Hobaby contributed to conceptualization, data curation, formal analysis, investigation, methodology, project administration, resources, software, supervision, validation, visualization, writing the original draft, review, and editing. Ali Etemadi contributed to formal analysis, supervision, validation, methodology, review, and editing. Mehrdad Mahalleh contributed to data curation, formal analysis, software, validation, writing – review, and editing. Maryam Taheri contributed to data curation, formal analysis, software, validation, writing – review, and editing. Adrian V. Hernandez contributed to the original data curation, project administration, software, and writing draft, review and editing. Toshiki Kuno contributed to writing, reviewing, and editing. Homa Taheri contributed to providing resources for review and editing. Robert J. Siegel contributed to validation, review, and editing. Florian Rader and Mohammad Hossein Mandegar contributed to formal analysis, project administration, providing resources, supervision, validation, review, and editing. Behnam N. Tehrani contributed to methodology, supervision, validation, writing, review, and editing. All authors have approved the final version of the manuscript.

## Ethics Statement

The authors have nothing to report.

## Conflicts of Interest

The authors declare no conflicts of interest.

## Supporting information

Supporting information.

Supporting information.

## Data Availability

Most of the data generated or analyzed during this study are included in this published article and its Supporting Information. Other data sets associated with the current study are available from the corresponding author upon reasonable request.
