# Supplementary material for: Renal Denervation Effects on Blood Pressure in Resistant and Uncontrolled Hypertension: A Meta‐Analysis of Sham‐Controlled Randomized Clinical Trials
Source: Clin Cardiol. 2025 Mar 1;48(3):e70104. doi: 10.1002/clc.70104 (PMC11871512; doi:10.1002/clc.70104)
Supplement: Supplementary file 1 — Supporting information. [file CLC-48-e70104-s001.docx]

***Renal Denervation effects on blood pressure in resistant and uncontrolled hypertension: a Systematic Review and Meta-Analysis of sham-controlled randomized clinical trials***

***Supplemental Figures***

***Quality assessment***

***Supplementary Figure 1:*** *Methodological Quality Summary Using RoB2*

***Supplementary Figure 2:*** *Effect of RDN vs. medical treatment night systolic and diastolic blood pressure*

***Supplementary Figure 3:*** *Effect of RDN vs. medical treatment on home systolic and diastolic blood pressure*

***Supplementary Figure 4:*** *Effect of RDN vs. medical treatment on daytime systolic and diastolic blood pressure*

***Supplementary Figure 5:*** *Effect of RDN vs. medical treatment on the* number of anti-hypertensive medications

***Supplementary Figure 6:*** *Effect of RDN vs. medical treatment on the complications and adverse event rate*

***Sensitivity and subgroup analyses* .................................................................................................................................**

***Supplementary Figure 7.*** *24-hour SBP, Fixed-Effect Model and Random-Effects Model, leave-one-out method*

***Supplementary Figure 8.*** *24-hour SBP, Random-Effects Model with Sample Size Threshold method.*

***Supplementary Figure 9.*** *24-hour SBP. Subgroup analysis by Device Type*

***Supplementary Figure 10.*** *24-hour SBP, Subgroup analyses by Medication Status*

***Supplementary Figure 11.*** *24-hour DBP, Fixed-Effect Model and Random-Effects Model, leave-one-out method*

***Supplementary Figure 12.*** *24-hour DBP, Random-Effects Model with Sample Size Threshold*

***Supplementary Figure 13.*** *Effect of renal denervation vs. medical treatment on 24-hour diastolic blood pressure stratified by type of intervention*

***Supplementary Figure 14.*** *Effect of renal denervation vs. medical treatment on 24-hour diastolic blood pressure stratified by medication status*

***Supplementary Figure 15.*** *Effect of renal denervation vs. medical treatment on office systolic blood pressure using Fixed-Effect Model and Random-Effects Model, leave-one-out method*

***Supplementary Figure 16.*** *Effect of renal denervation vs. medical treatment on office systolic blood pressure using a random-effects model with Sample Size Threshold method*

***Supplementary Figure 17.*** *Effect of renal denervation vs. medical treatment on office systolic blood pressure stratified by type of intervention*

***Supplementary Figure 18.*** *Effect of renal denervation vs. medical treatment on office diastolic blood pressure using Fixed-Effect Model and Random-Effects Model, leave-one-out method*

***Supplementary Figure 19.*** *Effect of renal denervation vs. medical treatment on office diastolic blood pressure using a random-effects model with Sample Size Threshold method.*

***Supplementary Figure 20.*** *Effect of renal denervation vs. medical treatment on office diastolic blood pressure stratified by type of intervention*

***Supplementary Figure 21.*** *Effect of renal denervation vs. medical treatment on home systolic blood pressure using Fixed-Effect Model and Random-Effects Model, leave-one-out method*

***Supplementary Figure 22.*** *Effect of renal denervation vs. medical treatment on home diastolic blood pressure using Fixed-Effect Model and Random-Effects Model, leave-one-out method*

***Supplementary Figure 23.*** *Effect of renal denervation vs. medical treatment on night systolic blood pressure using Fixed-Effect Model and Random-Effects Model, leave-one-out method*

***Supplementary Figure 24.*** *Effect of renal denervation vs. medical treatment on night systolic blood pressure stratified by type of intervention*

***Supplementary Figure 25.*** *Effect of renal denervation vs. medical treatment on night diastolic blood pressure using Fixed-Effect Model and Random-Effects Model, leave-one-out method*

***Supplementary Figure 26.*** *Effect of renal denervation vs. medical treatment on night diastolic blood pressure stratified by type of intervention*

***Supplementary Figure 27.*** *Effect of renal denervation vs. medical treatment on day systolic blood pressure using Fixed-Effect Model and Random-Effects Model, leave-one-out method*

***Supplementary Figure 28.*** *Effect of renal denervation vs. medical treatment on day systolic blood pressure stratified by type of intervention*

***Supplementary Figure 29.*** *Effect of renal denervation vs. medical treatment on day diastolic blood pressure using Fixed-Effect Model and Random-Effects Model, leave-one-out method*

***Supplementary Figure 30.*** *Effect of renal denervation vs. medical treatment on day diastolic blood pressure stratified by type of intervention*

***Supplementary Figure 31.*** *Effect of renal denervation vs. medical treatment on the number of anti-hypertensive medications using Fixed-Effect Model and Random-Effects Model, leave-one-out method*

***Supplementary Figure 32.*** *Effect of renal denervation vs. medical treatment on the number of anti-hypertensive medications using a random-effects model with Sample Size Threshold method.*

***Supplementary Figure 33.*** *Effect of renal denervation vs. medical treatment on drug index using Fixed-Effect Model and Random-Effects Model, leave-one-out method*

***Meta-regression analyses* ..............................................................................................................................**

***Supplementary Figure 34.*** *Meta-regression analysis evaluating age, body mass index (BMI), the proportion of male patients, glomerular filtration rate (GFR), baseline mean SBP, and follow-up time as covariates in 24-hour systolic and diastolic blood pressure.*

***Publication Bias ............................................................................................................................***

***Supplementary Figure 35.*** *Comparison-adjusted funnel plot for included studies that report the number of anti-hypertensive medications.*

***Effect of Time on Renal Denervation Efficacy***

***Supplementary Figure 36:*** *The time trend analysis of the three studies with 2-12-month follow-up*

***Quality Assessment***

A:


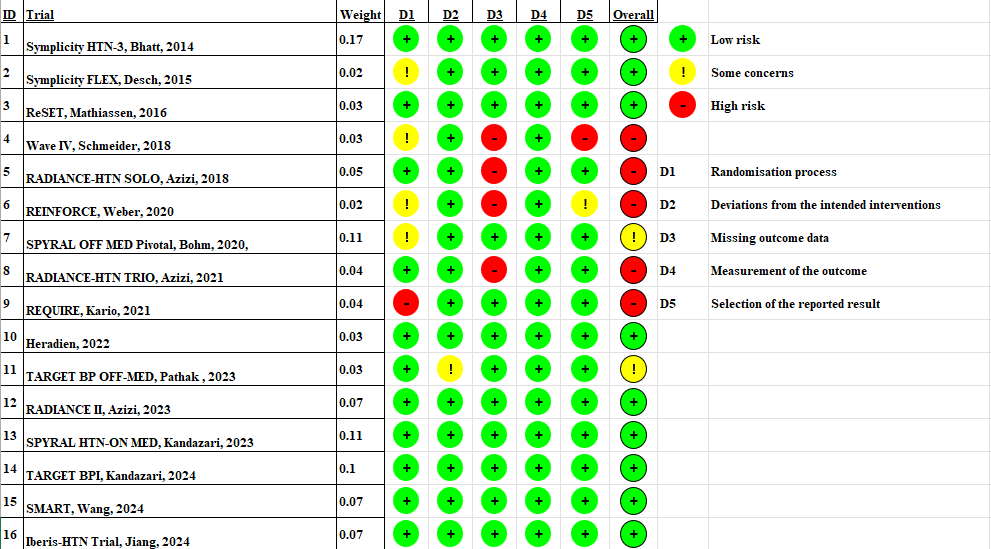


B:
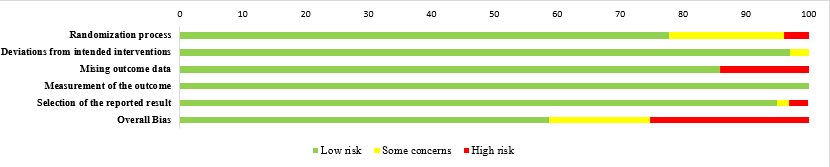


***Supplementary Figure 1.*** *Methodological quality summary using RoB2.* ***A.*** *The Risk of Bias Domains.* ***B*** *Overall risk of bias*

***Secondary outcome analyses***

***
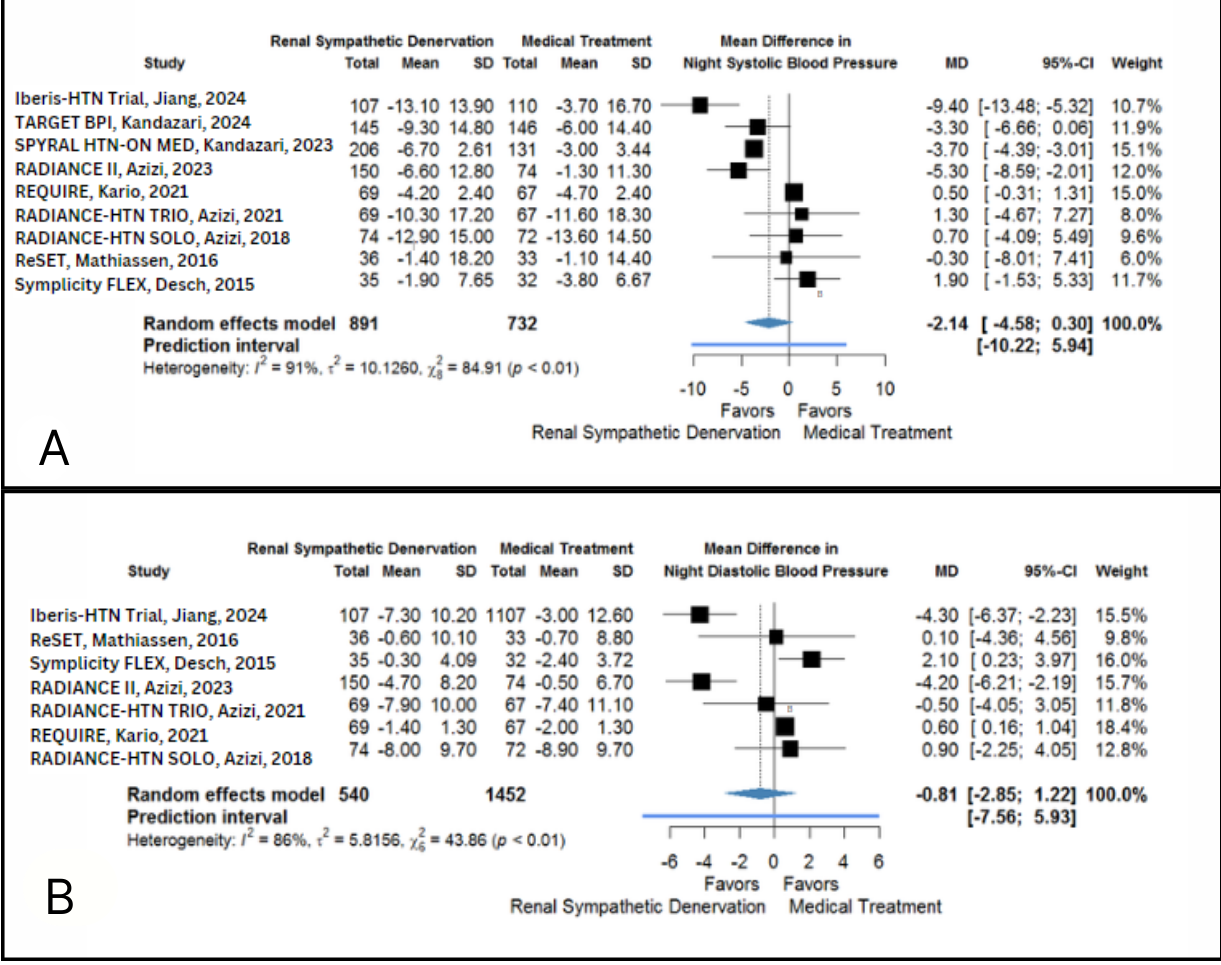
***


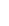


***Supplementary Figure 2:*** *Effect of RDN vs. medical treatment night systolic and diastolic blood pressure*

*
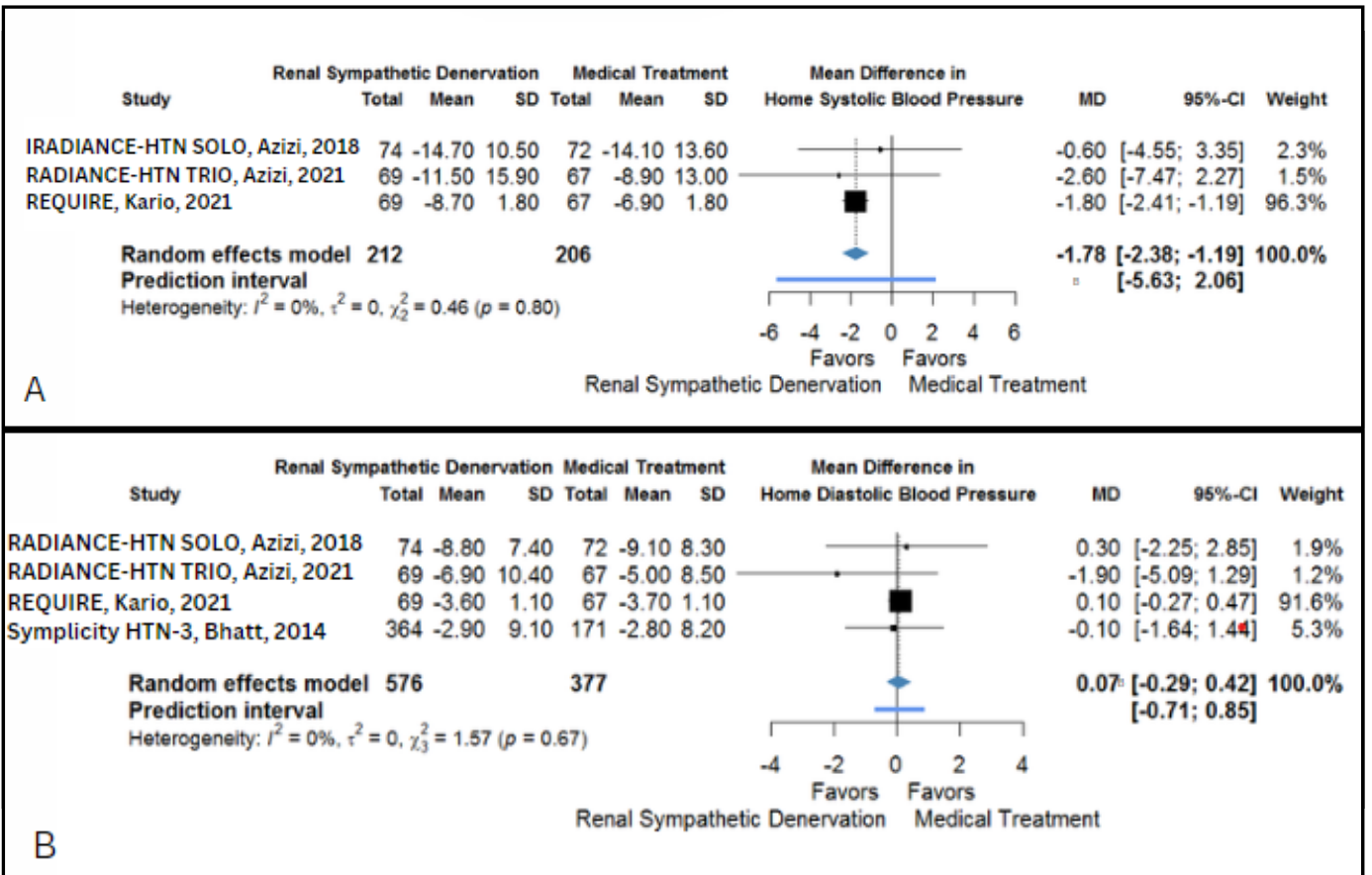
*

***Supplementary Figure 3:*** *Effect of RDN vs. medical treatment on home A. systolic and B. diastolic blood pressure*

***
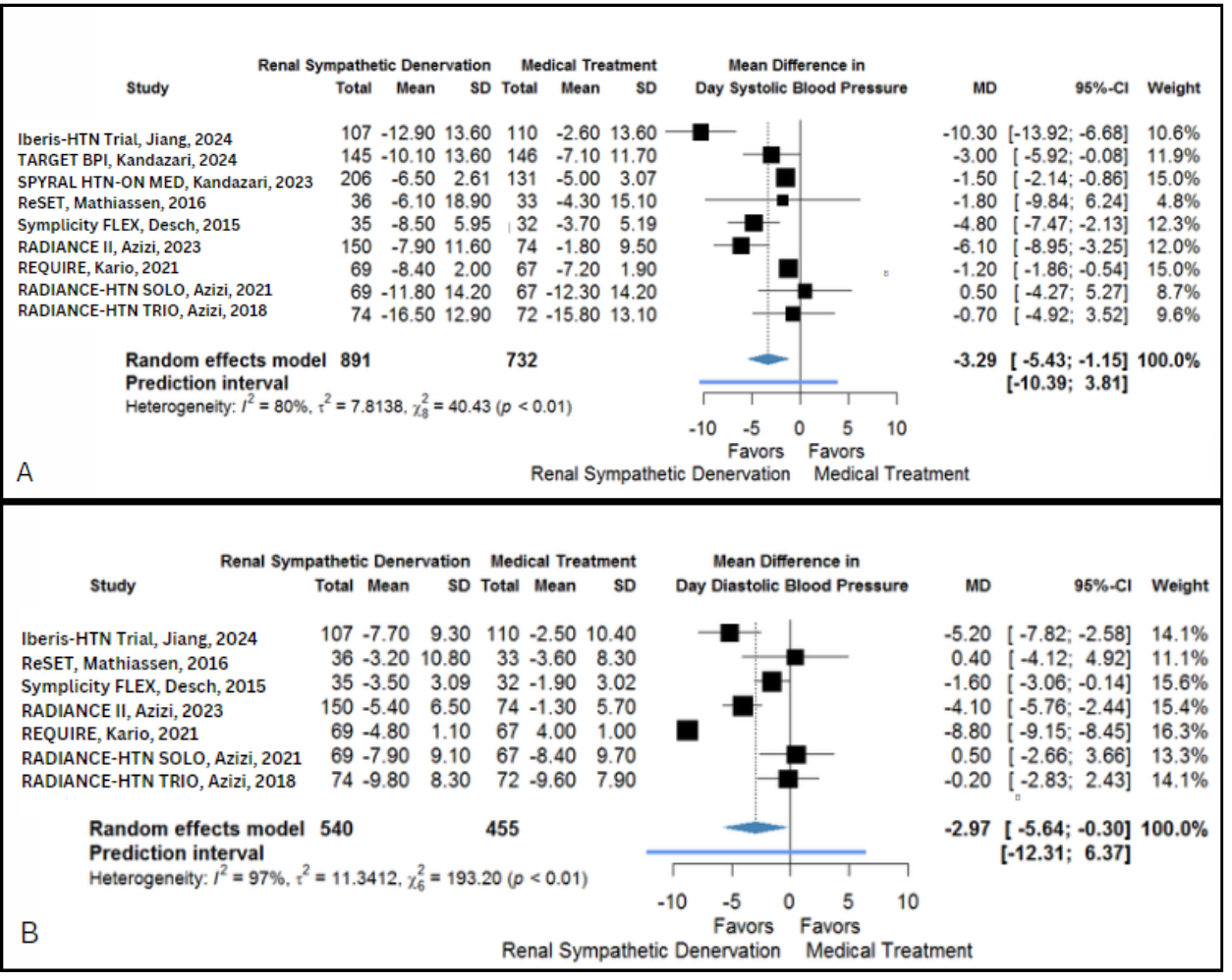
***

***Supplementary Figure 4:*** *Effect of RDN vs. medical treatment on daytime A. Systolic B. Diastolic blood pressure*

***
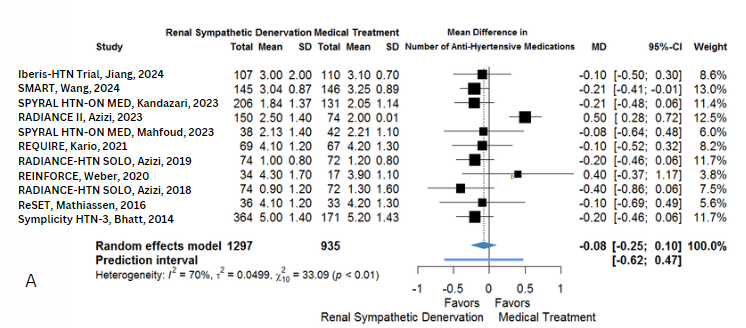
***

***Supplementary Figure 5:*** *Effect of RDN vs. medical treatment on the* number of anti-hypertensive medications

***
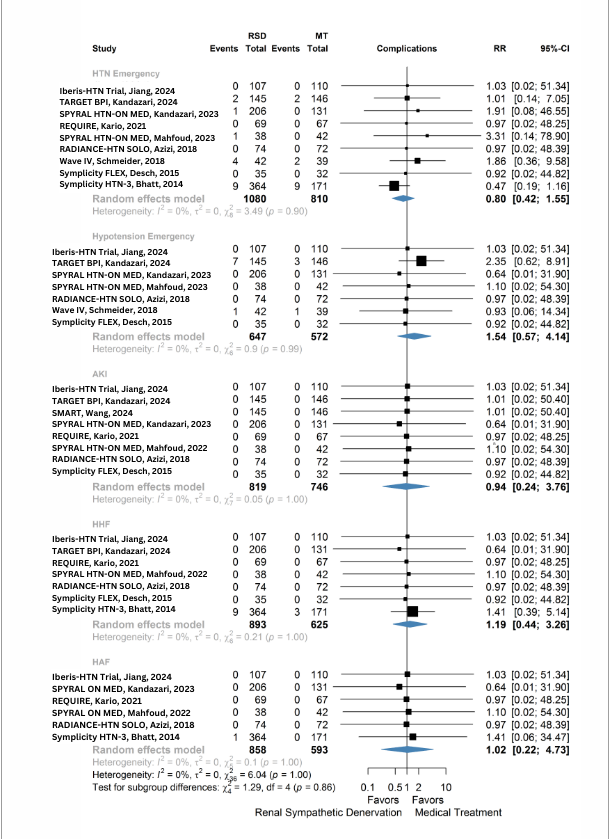

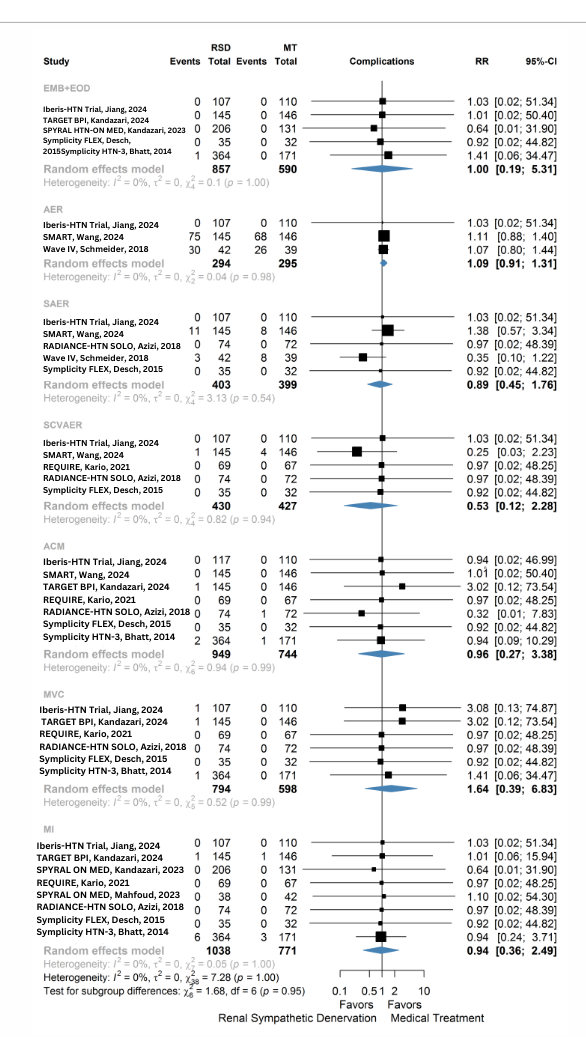
***

***
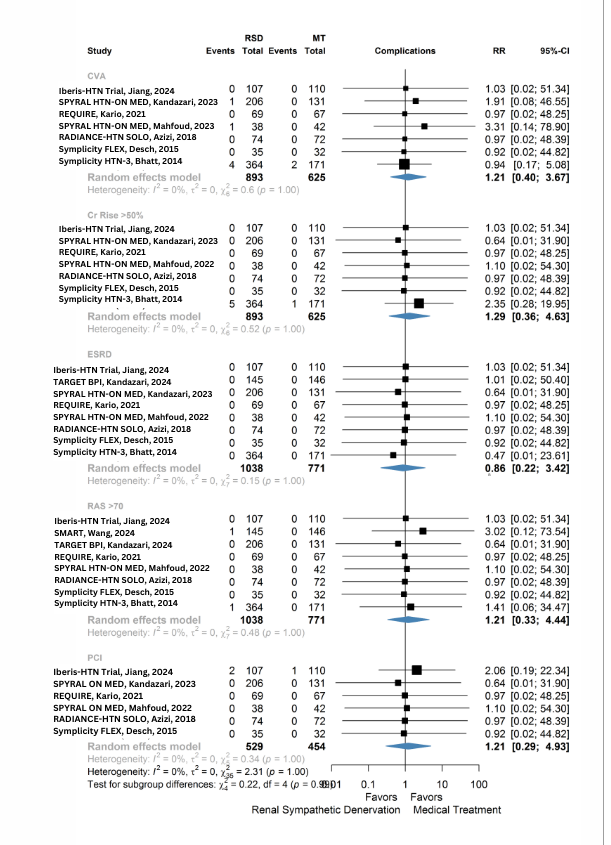
***

***Supplementary Figure 6:*** *Effect of RDN vs. medical treatment on the complications and adverse event rate*

***Sensitivity and subgroup analyses***

- ***24-hour ambulatory mean of systolic blood pressure***

**
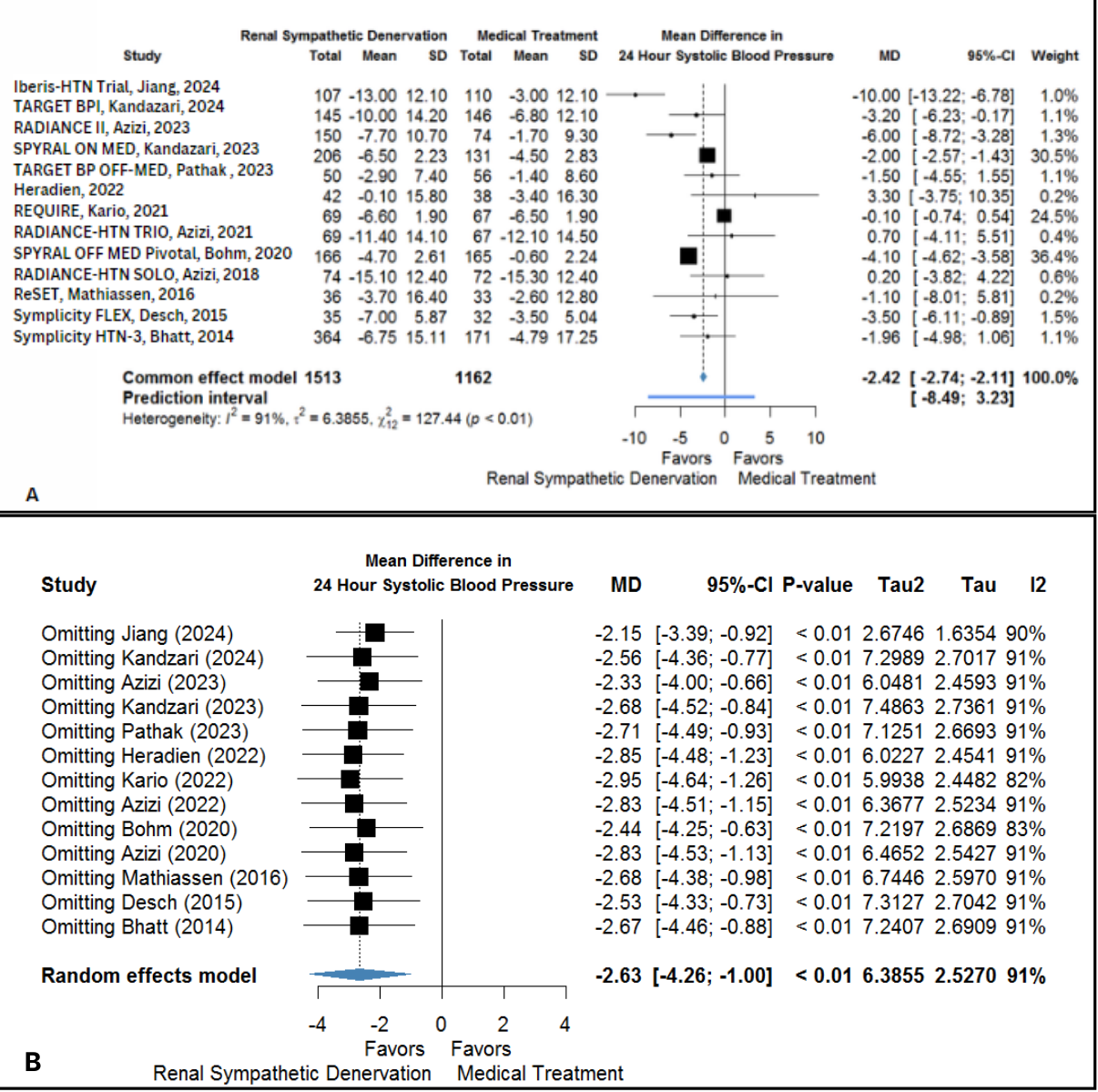
**

***Supplementary Figure 7.*** *Effect of renal denervation vs. medical treatment on 24-hour systolic blood pressure using* ***A****. fixed-effect model,* ***B.*** *random-effects model, and the leave-one-out method.*

***
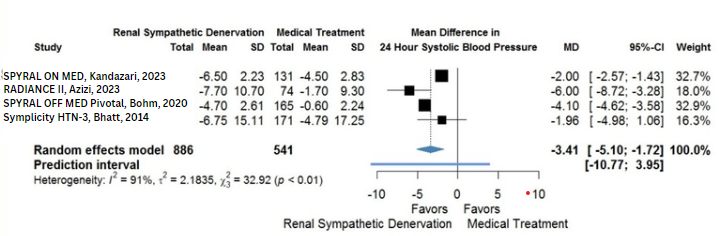
***

***Supplementary Figure 8.*** *Effect of renal denervation vs. medical treatment on 24-hour systolic blood pressure using a random-effects model with the inclusion of studies of 100 or more individuals.*


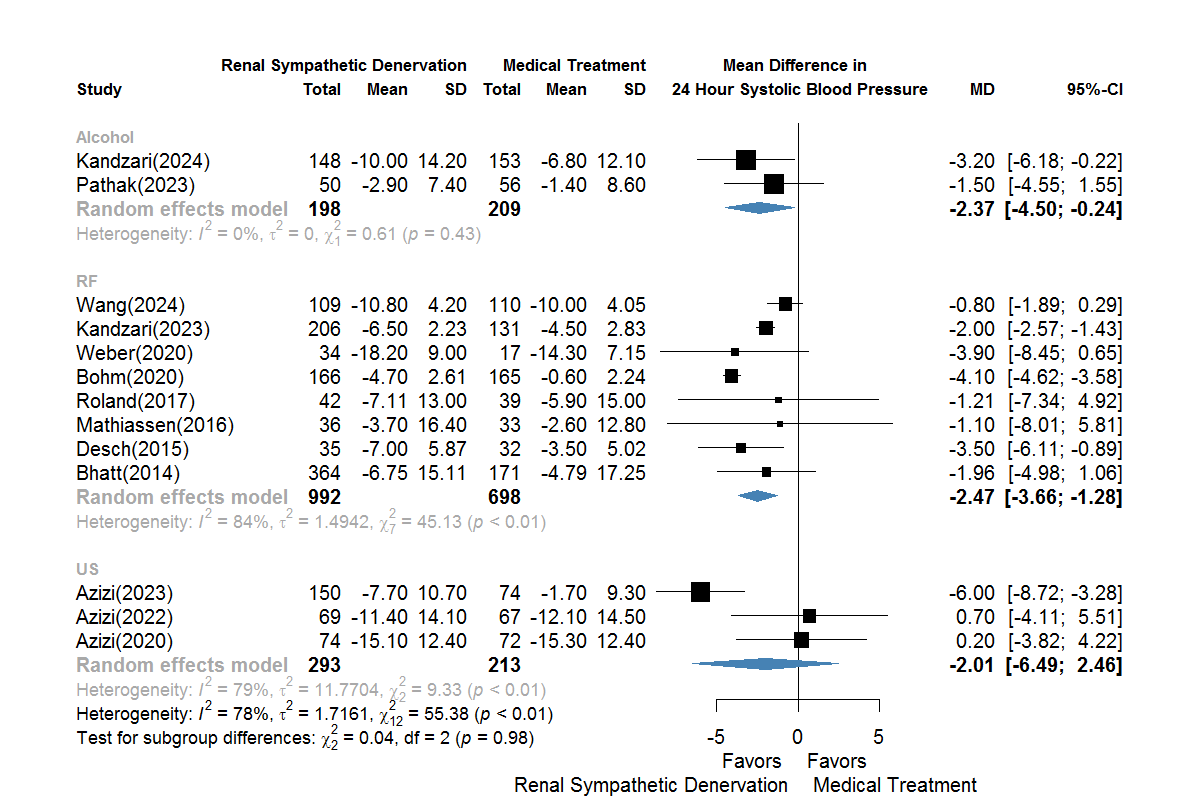


***Supplementary Figure 9.*** *Effect of renal denervation vs. medical treatment on 24-hour systolic blood pressure stratified by type of intervention: Alcohol, Radiofrequency, and Ultrasound.*


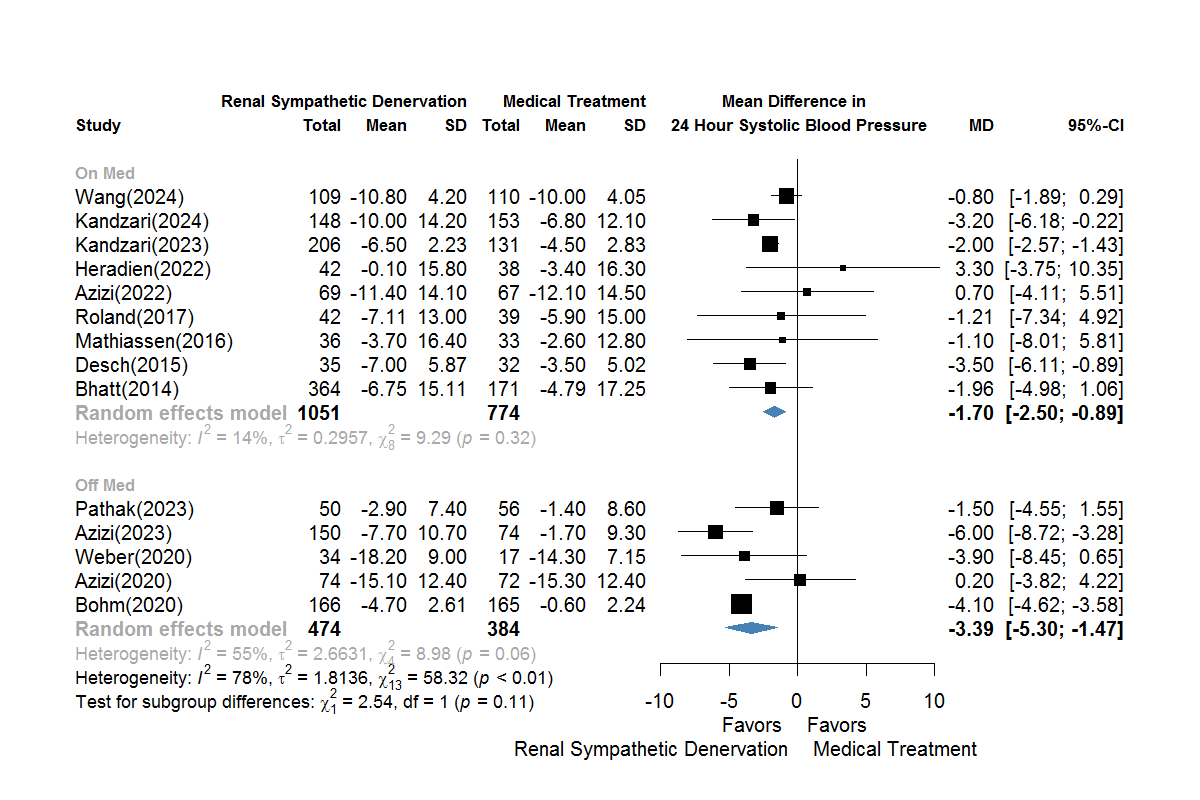


***Supplementary Figure 10.*** *Effect of renal denervation vs. medical treatment on 24-hour systolic blood pressure stratified by medication status: On Med (patients on medication) and Off Med (patients off medication).*

- ***24-hour ambulatory mean of diastolic blood pressure***

**
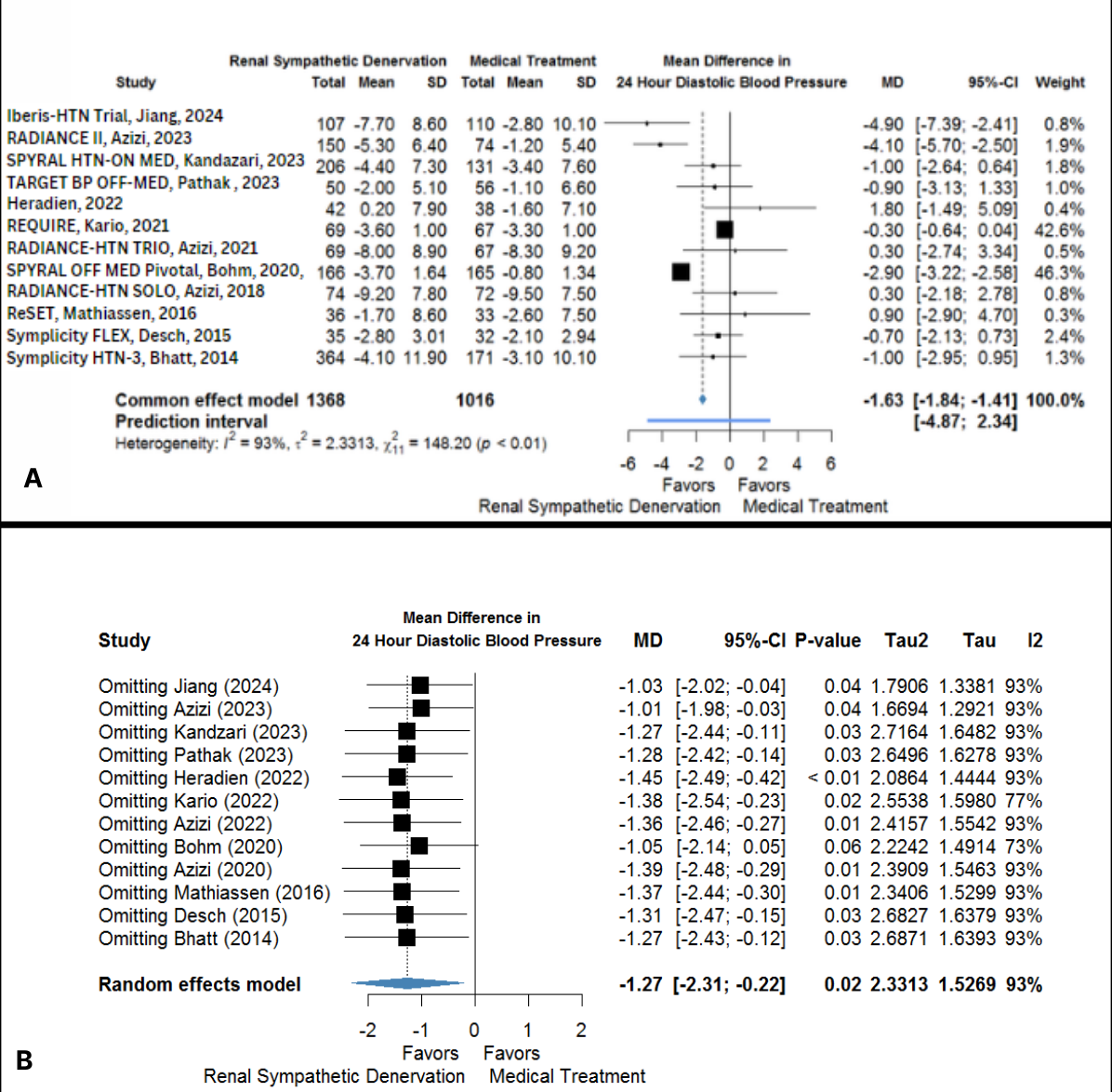
**

***Supplementary Figure 11.*** *Effect of renal denervation vs. medical treatment on 24-hour diastolic blood pressure using* ***A****. fixed-effect model,* ***B.*** *random-effects model, and the leave-one-out method.*


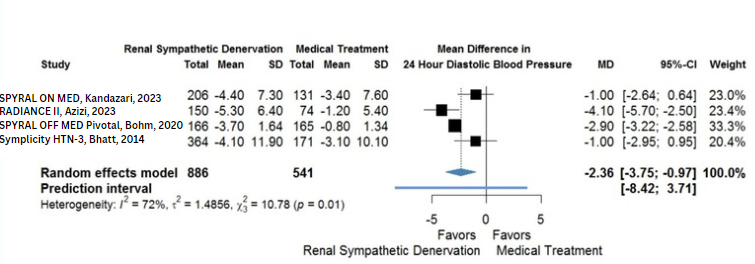


***Supplementary Figure 12.*** *Effect of renal denervation vs. medical treatment on 24-hour diastolic blood pressure using a random-effects model with the inclusion of studies of 100 or more individuals.*


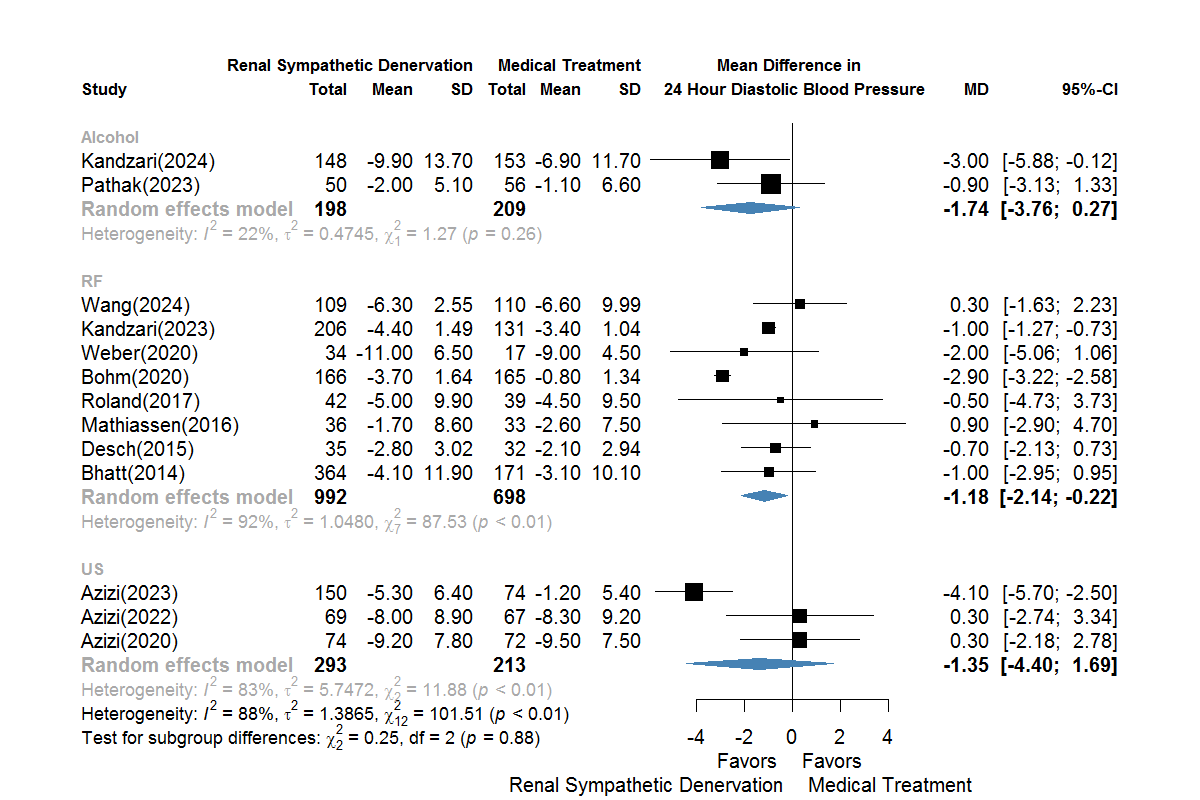


***Supplementary Figure 13.*** *Effect of renal denervation vs. medical treatment on 24-hour diastolic blood pressure stratified by type of intervention: Alcohol, Radiofrequency, and Ultrasound.*

***
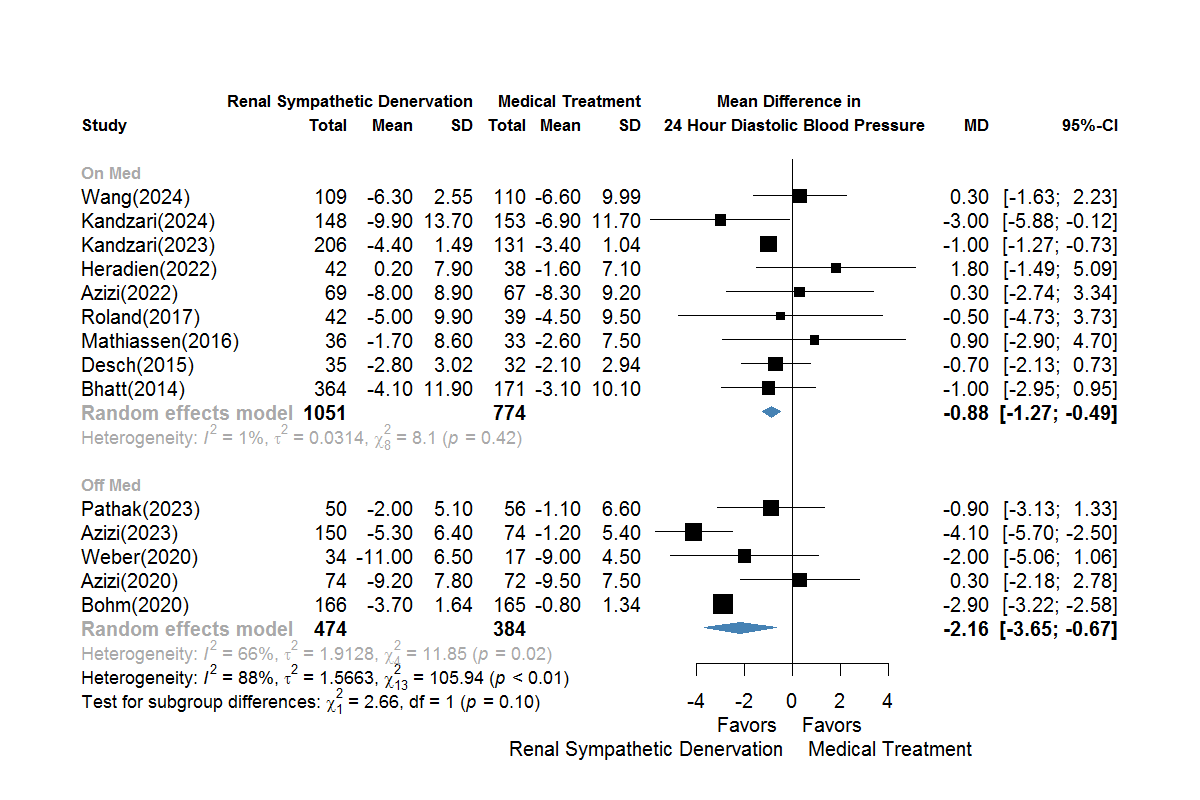
***

***Supplementary Figure 14.*** *Effect of renal denervation vs. medical treatment on 24-hour diastolic blood pressure stratified by medication status: On Med (patients on medication) and Off Med (patients off medication).*

- ***Office systolic blood pressure***

**
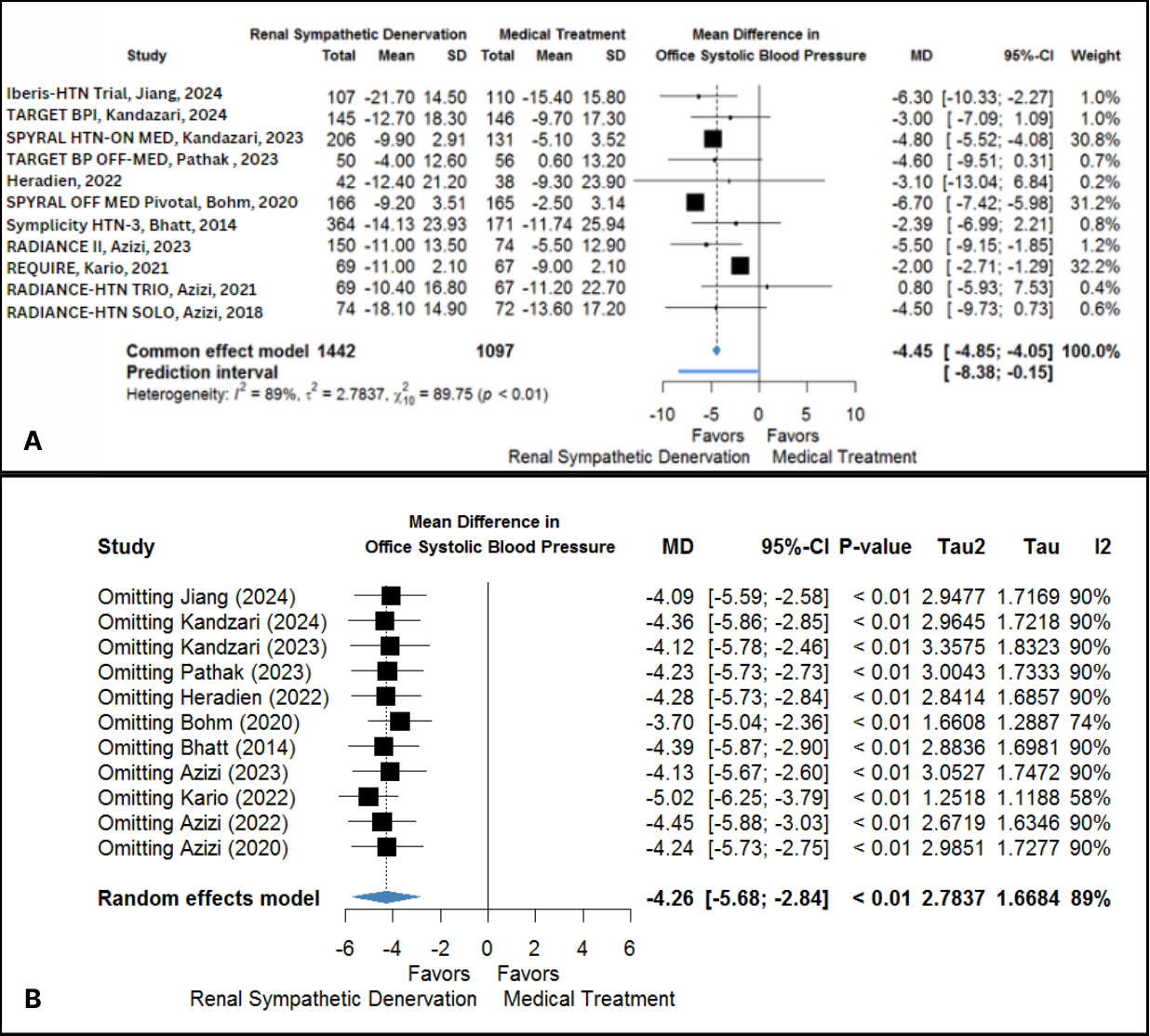
**

***Supplementary Figure 15.*** *Effect of renal denervation vs. medical treatment on office systolic blood pressure using* ***A****. fixed-effect model,* ***B.*** *random-effects model, and the leave-one-out method.*

**
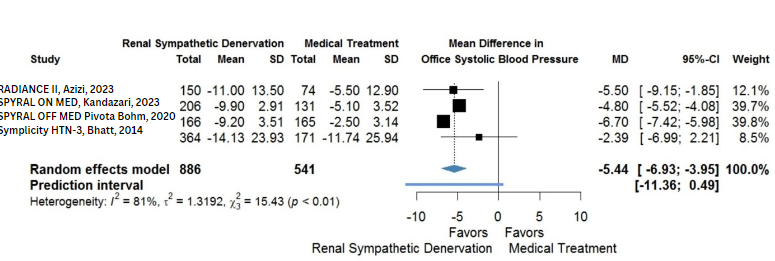
**

***Supplementary Figure 16.*** *Effect of renal denervation vs. medical treatment on office systolic blood pressure using a random-effects model with the inclusion of studies of 100 or more individuals.*

***
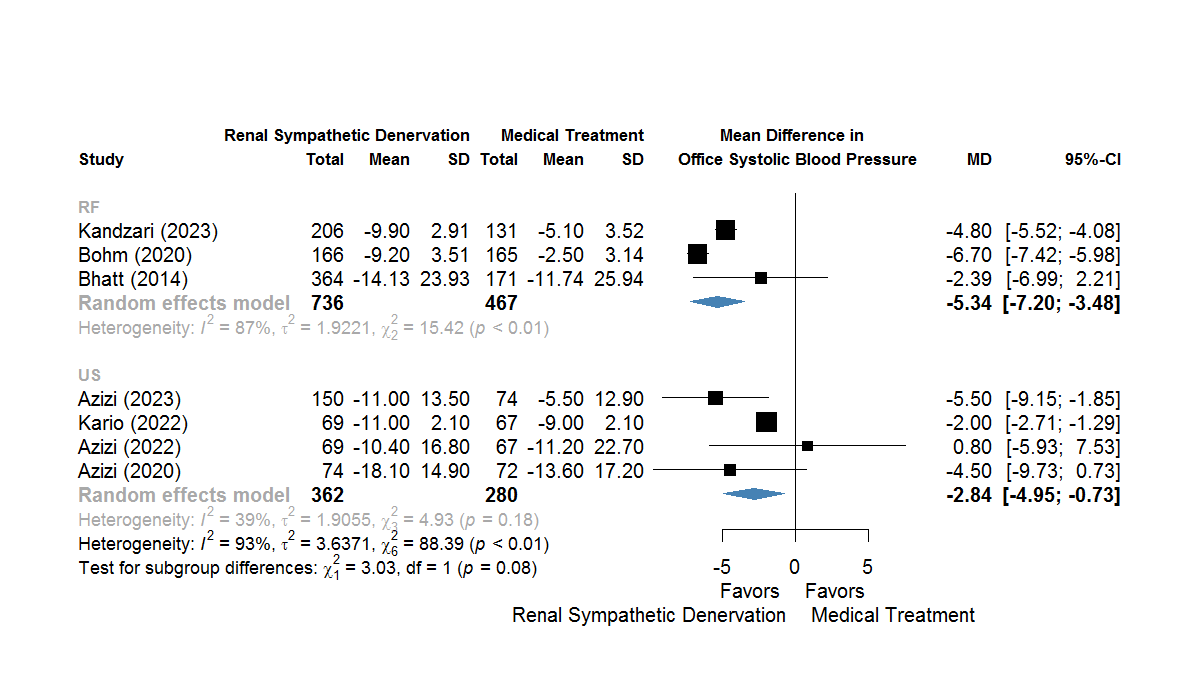
***

***Supplementary Figure 17.*** *Effect of renal denervation vs. medical treatment on office systolic blood pressure stratified by type of intervention: Radiofrequency and Ultrasound.*

- ***Office diastolic blood pressure***

**
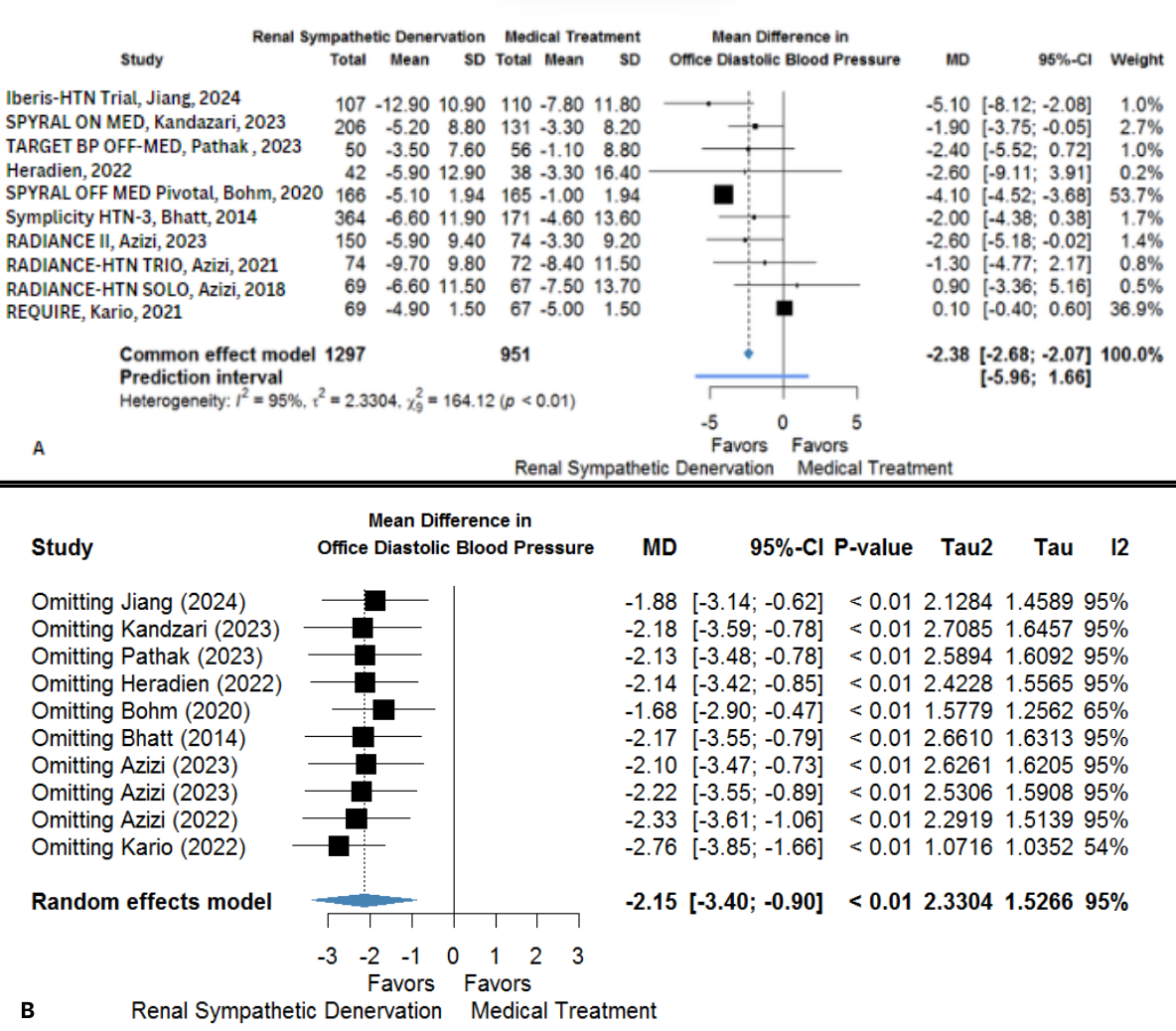
**

***Supplementary Figure 18.*** *Effect of renal denervation vs. medical treatment on office diastolic blood pressure using* ***A****. fixed-effect model,* ***B.*** *random-effects model, and the leave-one-out method.*


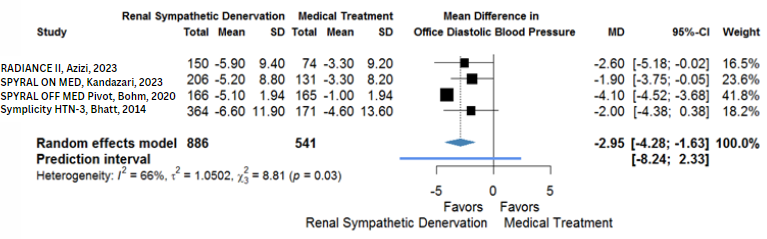


***Supplementary Figure 19.*** *Effect of renal denervation vs. medical treatment on office diastolic blood pressure using a random-effects model with the inclusion of studies of 100 or more individuals.*

*
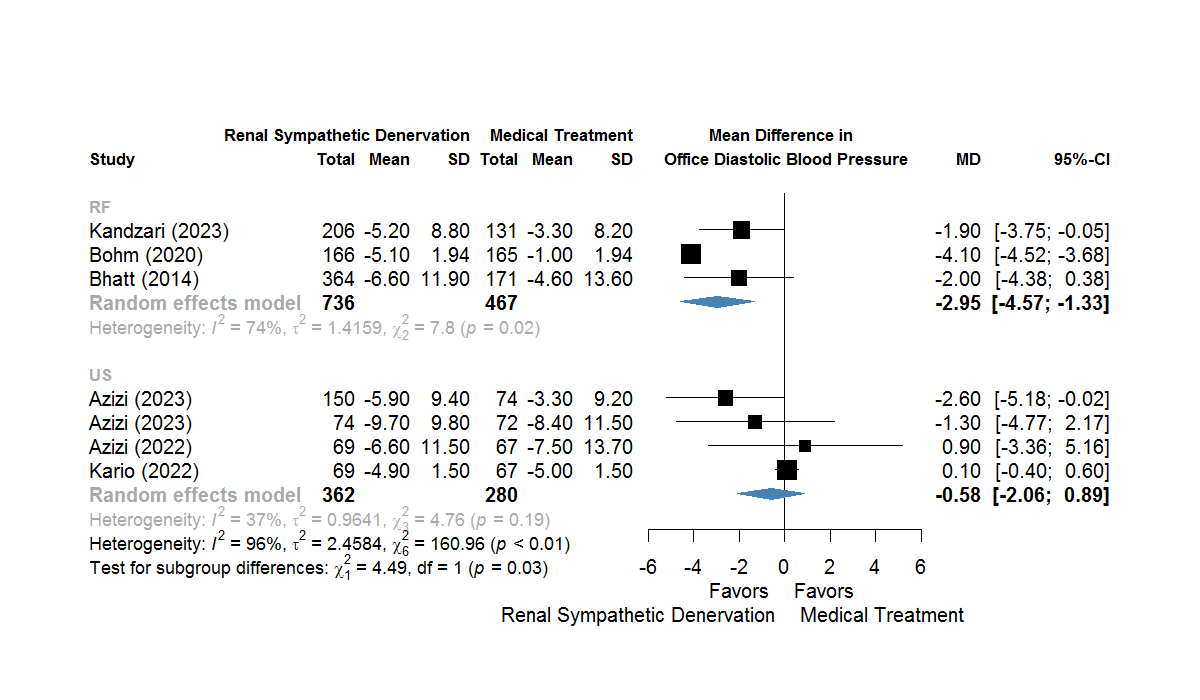
*

***Supplementary Figure 20.*** *Effect of renal denervation vs. medical treatment on office diastolic blood pressure stratified by type of intervention: Radiofrequency and Ultrasound.*

- ***Home systolic blood pressure***


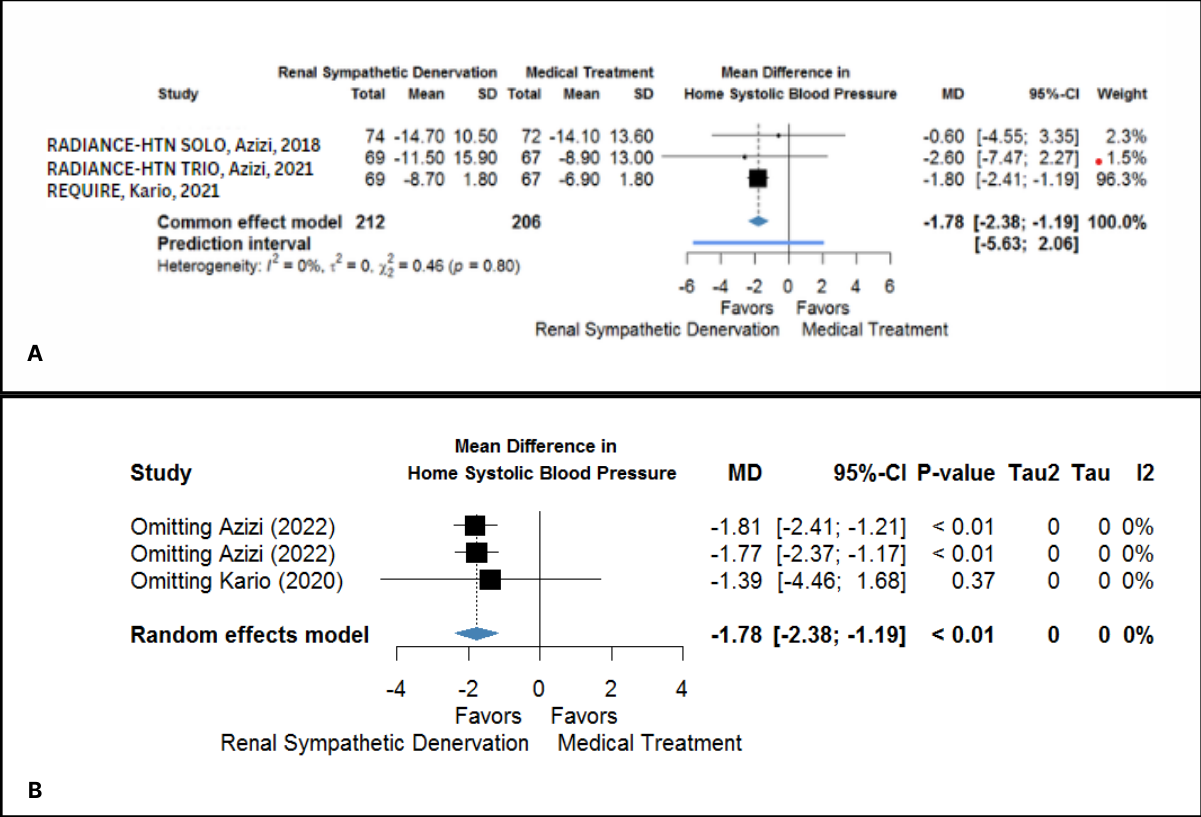


***Supplementary Figure 21.*** *Effect of renal denervation vs. medical treatment on home systolic blood pressure using* ***A****. fixed-effect model,* ***B.*** *random-effects model, and the leave-one-out method.*

- ***Home diastolic blood pressure***

**
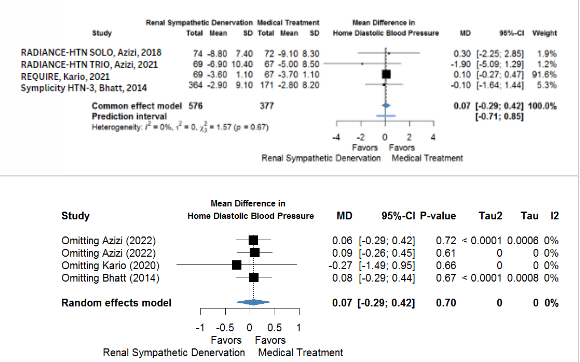
**

***Supplementary Figure 22.*** *Effect of renal denervation vs. medical treatment on home diastolic blood pressure using* ***A****. fixed-effect model,* ***B.*** *random-effects model, and the leave-one-out method.*

- ***Night systolic blood pressure***

**
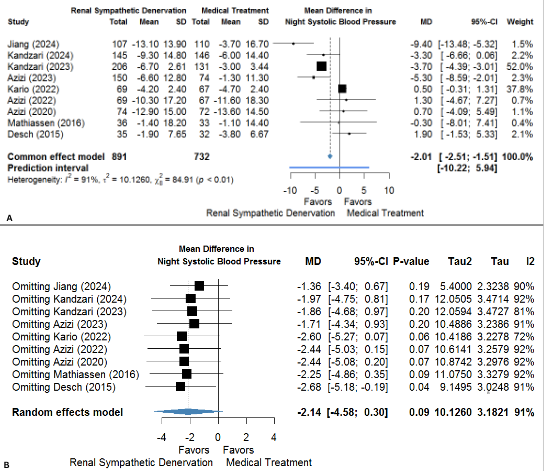
**

***Supplementary Figure 23.*** *Effect of renal denervation vs. medical treatment on night systolic blood pressure using* ***A****. fixed-effect model,* ***B.*** *random-effects model, and the leave-one-out method.*

***
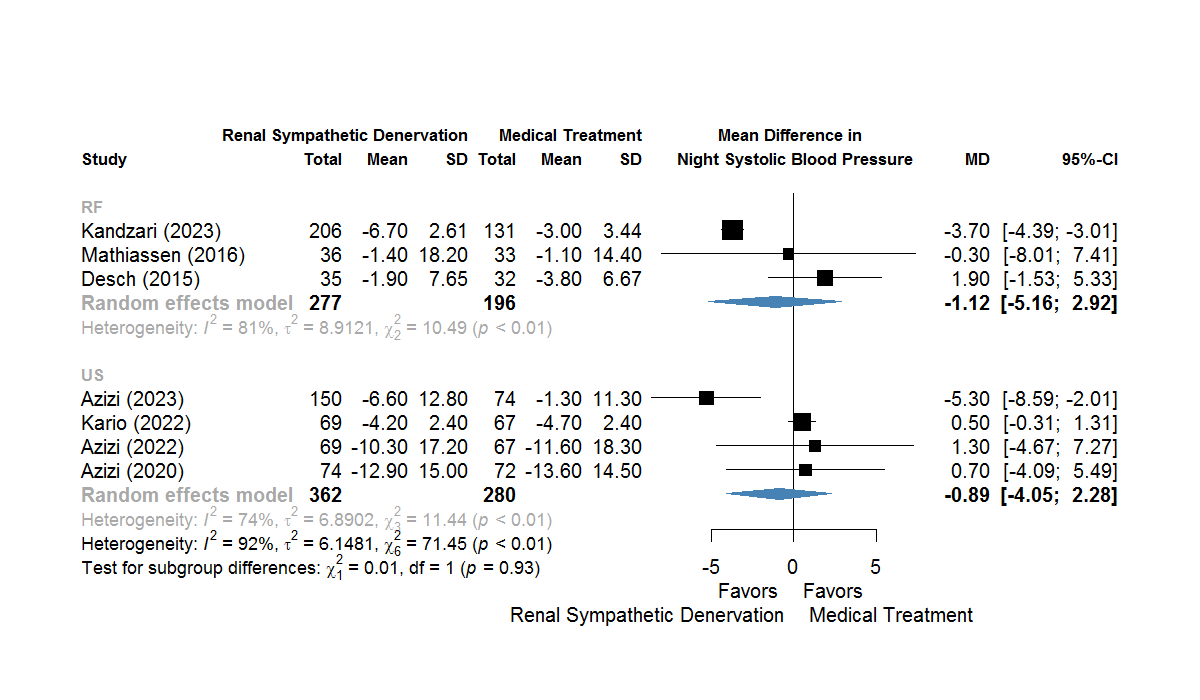
***

***Supplementary Figure 24.*** *Effect of renal denervation vs. medical treatment on night systolic blood pressure stratified by type of intervention: Radiofrequency and Ultrasound.*

- ***Night diastolic blood pressure***

**
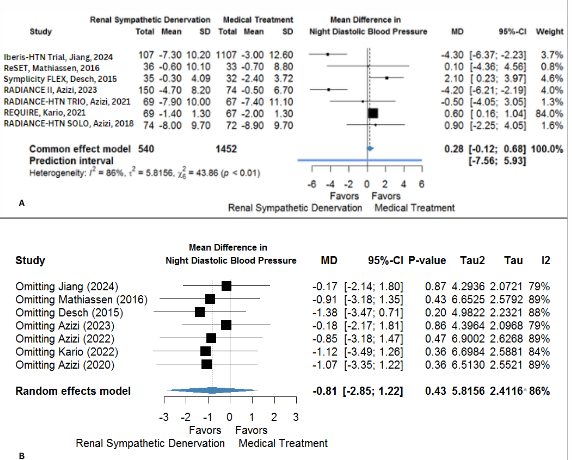
**

***Supplementary Figure 25.*** *Effect of renal denervation vs. medical treatment on night diastolic blood pressure using* ***A****. fixed-effect model,* ***B.*** *random-effects model, and the leave-one-out method.*


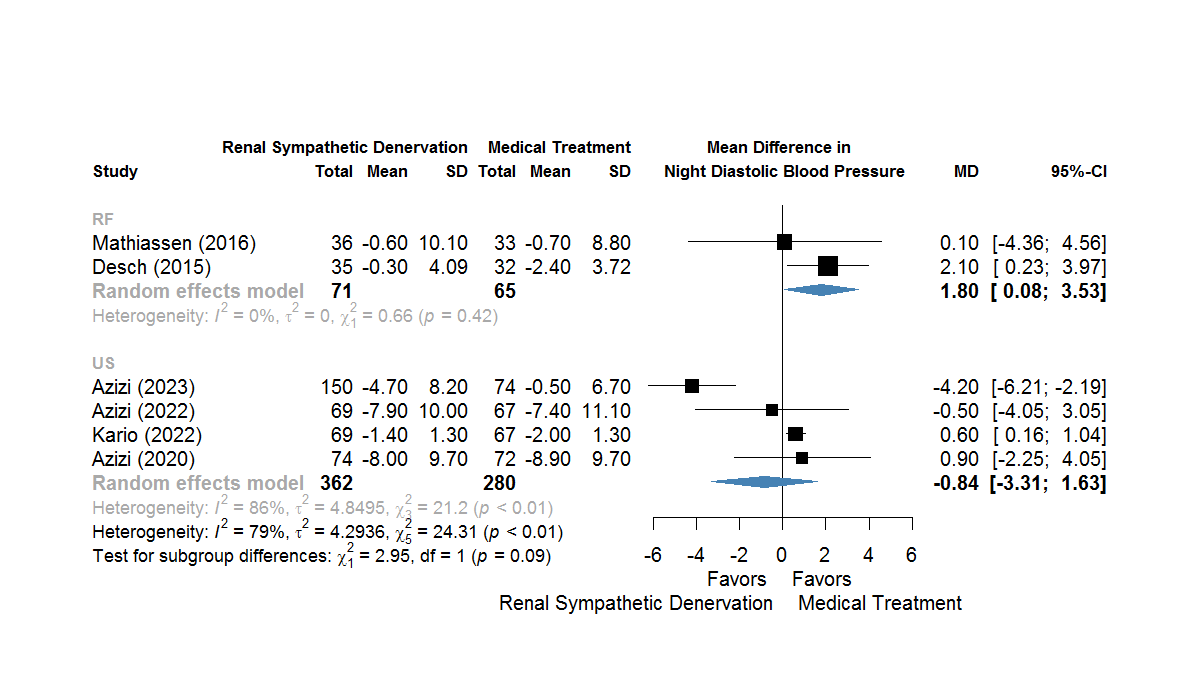


***Supplementary Figure 26.*** *Effect of renal denervation vs. medical treatment on night diastolic blood pressure stratified by type of intervention: Radiofrequency and Ultrasound.*

- ***Daytime systolic blood pressure***

**
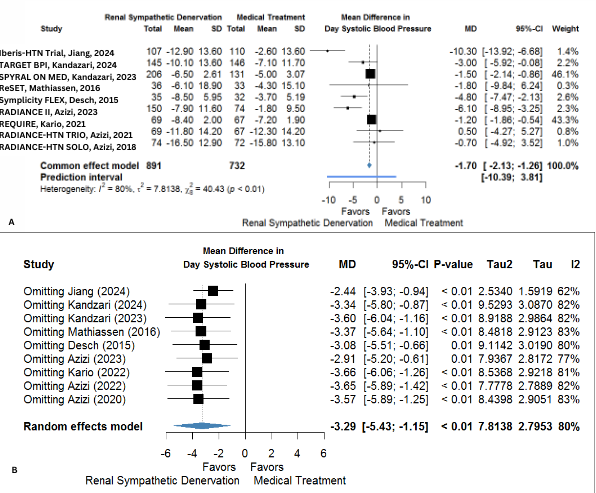
**

***Supplementary Figure 27.*** *Effect of renal denervation vs. medical treatment on day systolic blood pressure using* ***A****. fixed-effect model,* ***B.*** *random-effects model, and the leave-one-out method.*

***
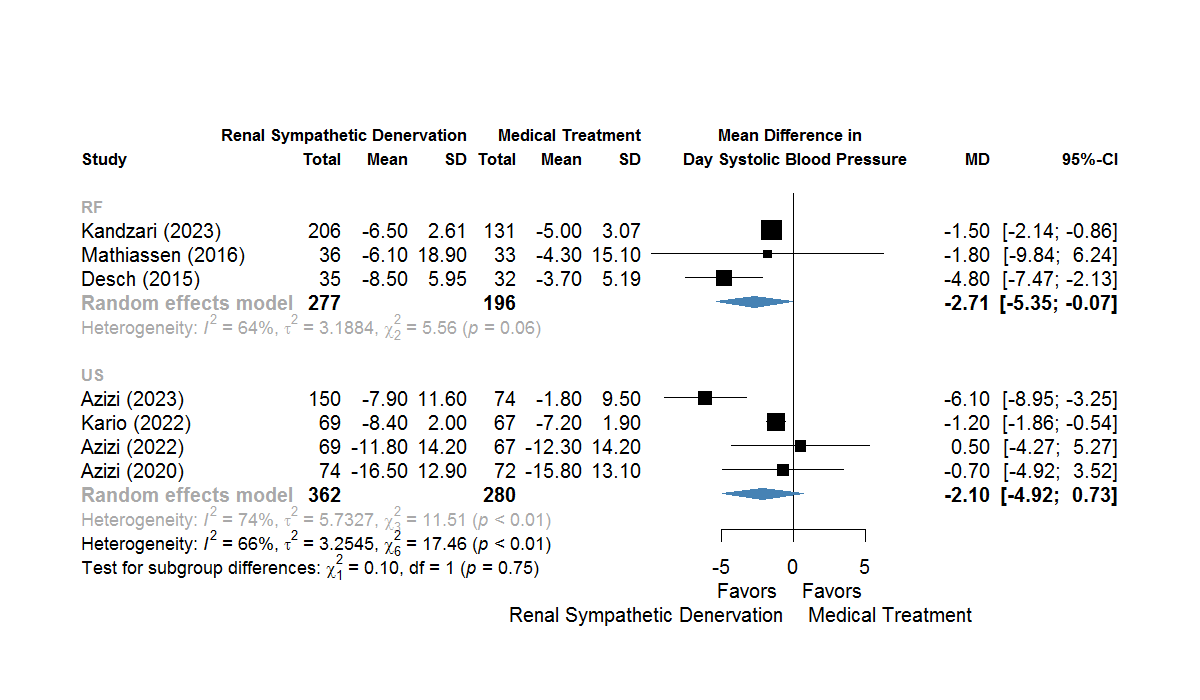
***

***Supplementary Figure 28.*** *Effect of renal denervation vs. medical treatment on day systolic blood pressure stratified by type of intervention: Radiofrequency and Ultrasound.*

- ***Daytime diastolic blood pressure***

**
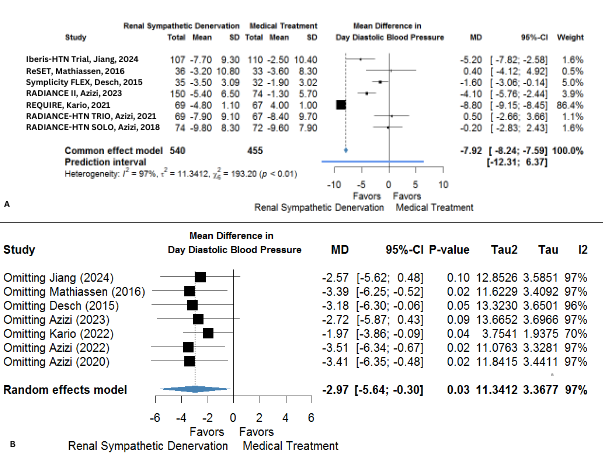
**

***Supplementary Figure 29.*** *Effect of renal denervation vs. medical treatment on day diastolic blood pressure using* ***A****. fixed-effect model,* ***B.*** *random-effects model, and the leave-one-out method.*

***
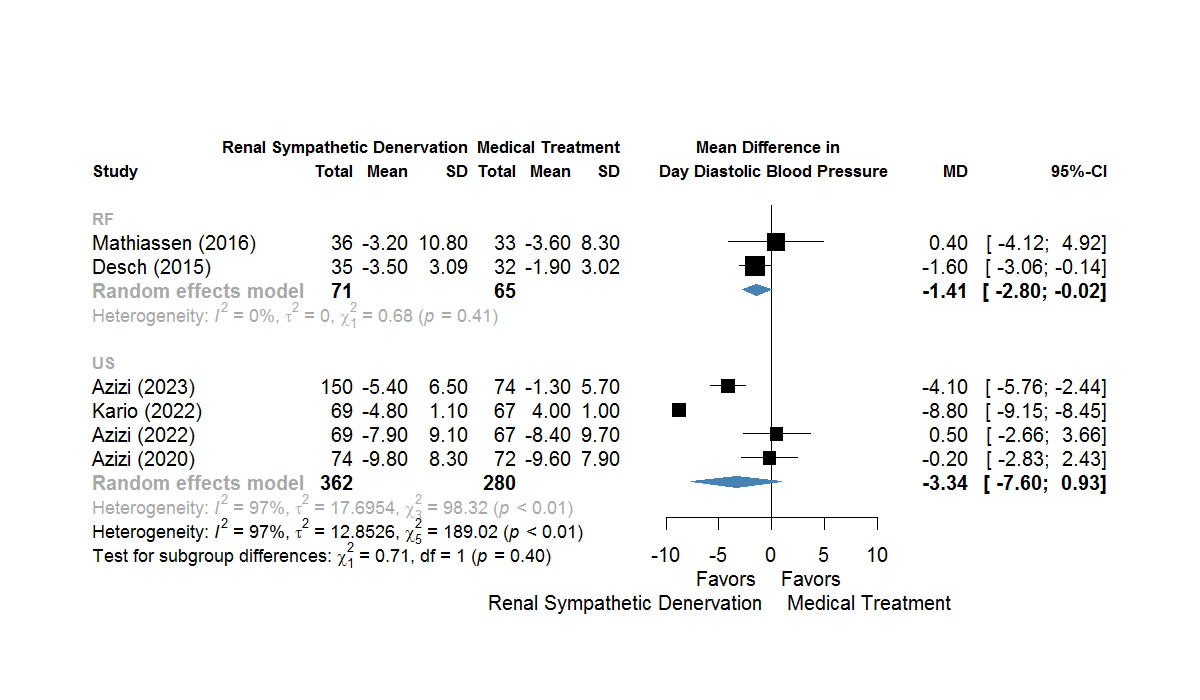
***

***Supplementary Figure 30.*** *Effect of renal denervation vs. medical treatment on day diastolic blood pressure stratified by type of intervention: Radiofrequency and Ultrasound.*

- ***Antihypertensive medications number***

**
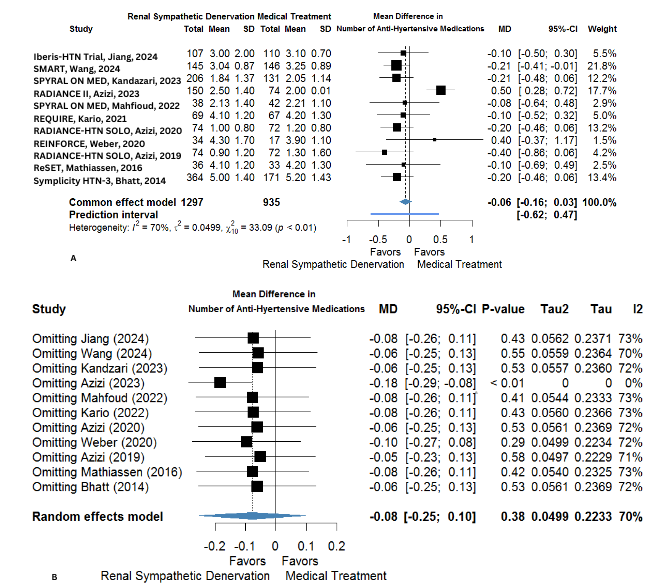
**

***Supplementary Figure 31.*** *Effect of renal denervation vs. medical treatment on the number of anti-hypertensive medications using* ***A****. fixed-effect model,* ***B.*** *random-effects model, and the leave-one-out method.*

**
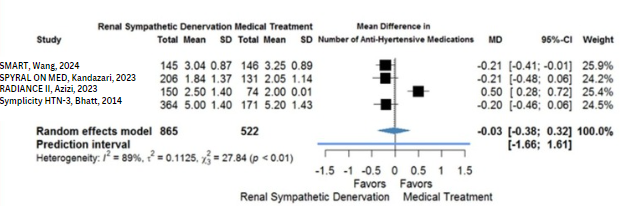
**

***Supplementary Figure 32.*** *Effect of renal denervation vs. medical treatment on the number of anti-hypertensive medications using a random-effects model with the inclusion of studies of 100 or more individuals.*

- ***Drug Index***

**
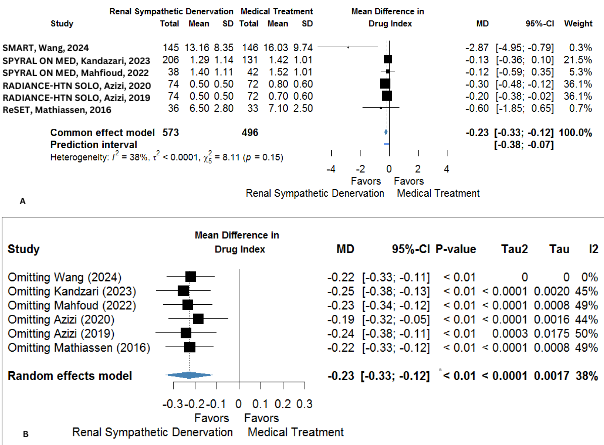
**

***Supplementary Figure 33.*** *Effect of renal denervation vs. medical treatment on drug index using* ***A****. fixed-effect model,* ***B.*** *random-effects model, and the leave-one-out method.*

***Meta-Regression***

**
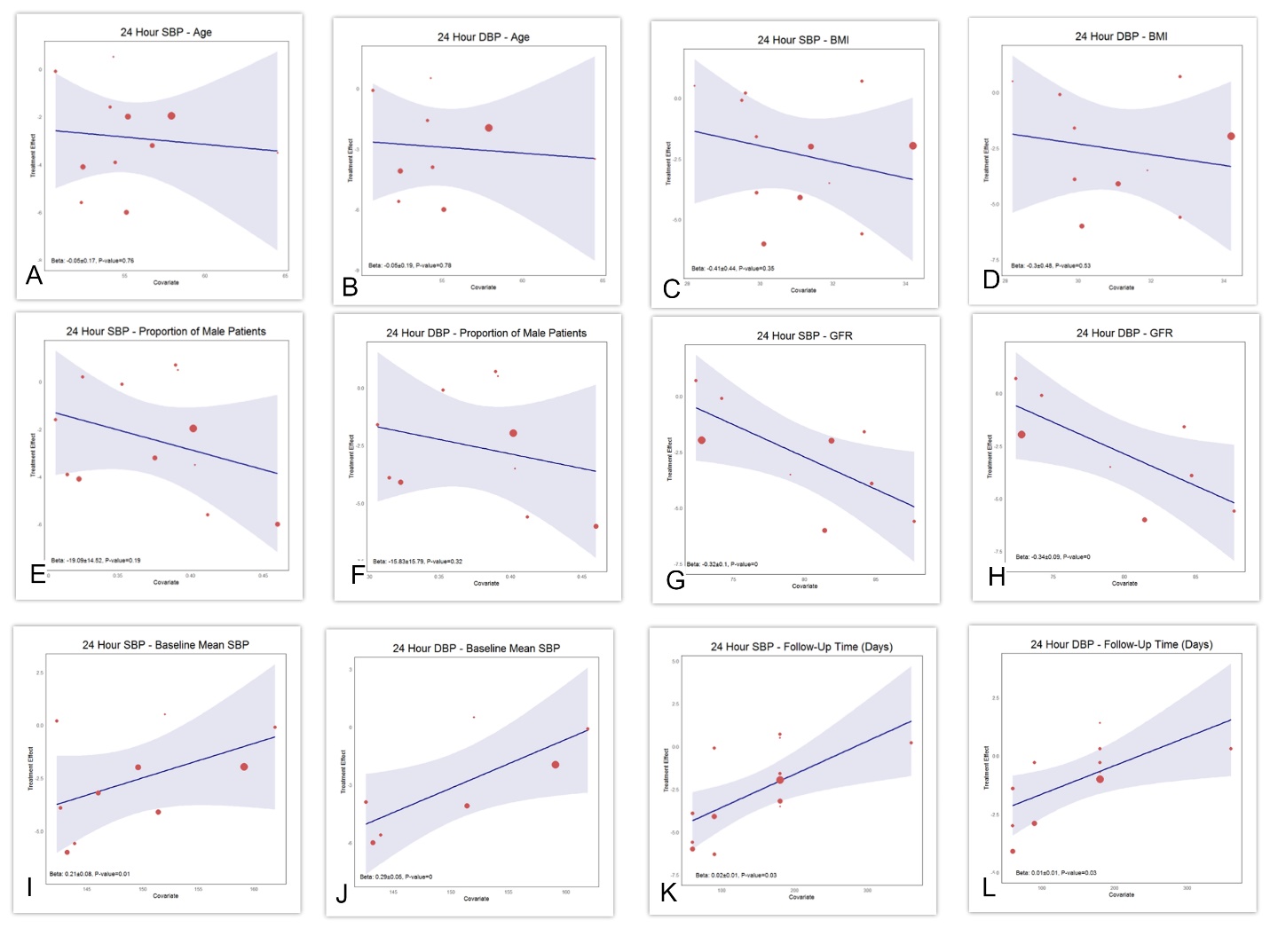
**

***Supplementary Figure 34.*** *Meta-regression analysis evaluating* age, body mass index (BMI), the proportion of male patients, glomerular filtration rate (GFR), baseline mean SBP, and follow-up time *as covariates in 24-hour systolic and diastolic blood pressure.*

***Publication Bios***

**
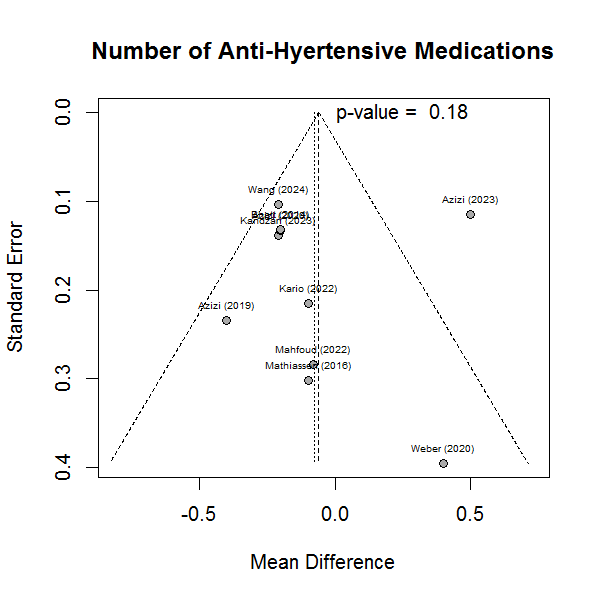
**

***Supplementary Figure 35.*** *Comparison-adjusted funnel plot for included studies that report the number of anti-hypertensive medications.*

***
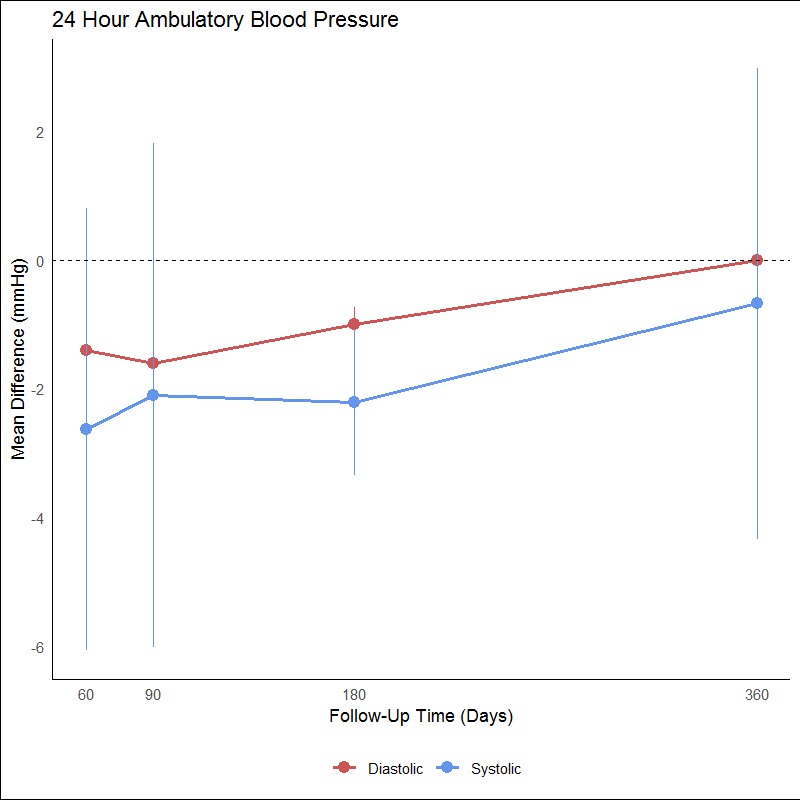
***

***Supplementary Figure 36:*** *The time trend analysis of the three studies with 2-12-month follow-up*
