# Supplementary material for: Renal Denervation Effects on Blood Pressure in Resistant and Uncontrolled Hypertension: A Meta‐Analysis of Sham‐Controlled Randomized Clinical Trials
Source: Clin Cardiol. 2025 Mar 1;48(3):e70104. doi: 10.1002/clc.70104 (PMC11871512; doi:10.1002/clc.70104)
Supplement: Supplementary file 2 — Supporting information. [file CLC-48-e70104-s002.docx]

**Supplementary Tables**

**Renal Denervation effects on blood pressure in resistant and uncontrolled hypertension: a Systematic Review and Meta-Analysis of sham-controlled randomized clinical trials**

**Supplementary Table 1.** Search query used for gathering data from databases

**Supplementary Table 2.** Summary of secondary analyses of outcomes

**Supplementary Table 3.** The comparison between the drug index and the average of anti-hypertension medication before and after the intervention and follow-up time.

**Supplementary Table 4**. Complications of Renal Denervation (RDN) and Sham Procedures

**Supplementary Table 5.** Comparison of Treatment Effects on Blood Pressure Outcomes Between Random Effects and Fixed Effects Models

**Supplementary table 1:** Search query used for gathering data from databases

| **Domain** | **Query** |
| --- | --- |
| **#1** | (HTN OR Hypertension OR “High BP” OR “High blood pressure” OR “elevated BP” OR “elevated blood pressure” OR “High systolic BP” OR “elevated systolic BP” OR “high systolic blood pressure” OR “elevated systolic blood pressure” OR “high SBP” OR “elevated SBP” OR “High diastolic BP” OR “elevated diastolic BP” OR “high diastolic blood pressure” OR “elevated diastolic blood pressure” OR “high DBP” OR “elevated DBP” OR “high Blood pressure level” OR “high BP level” OR “hypertensive disease” OR “HTN-hypertension” OR “uncontrolled blood pressure” OR “uncontrolled BP” OR "hypertension" OR "blood pressure" OR "hypertensive" OR hyperten* OR "high blood pressure" OR "elevated blood pressure" OR "abnormal blood pressure") |
| **#2** | ("controlled HTN" OR "controlled hypertension" OR "BP reduction" OR "Blood pressure reduction" OR "lowering BP" OR "lowering blood pressure" OR "antihypertensive therapy" OR "blood pressure management") |
| **#3** | ("cardiovascular morbidity" OR "cardiovascular mortality" OR "cardiovascular death" OR "cardiovascular outcome" OR  "cardiovascular outcomes" OR "cardiac outcome" OR  "cardiac outcomes" OR "cardiac arrest" OR "cardiac death" OR mortality OR death OR morbidity OR "Major Adverse Cardiovascular Event" OR "Major Adverse Cardiovascular Event" OR mace OR "Major Adverse Cardiac Event" OR "Major Adverse Cardiac Events" OR "cardiovascular events" OR "cardiac events" OR "ischemic heart disease" OR "heart failure" OR "major cardiovascular disease" OR "major CVD" OR cvd "HTN-mediated organ damage" OR "hypertension therapy" OR "antihypertensive therapy") |
| **#4** | ("renal denervation" OR rdn OR "renal sympathetic denervation" OR "renal nerve ablation" OR "renal sympathetic nerve" OR "renal sympathetic nerves" OR "renal sympathetic ablation" OR "radiofrequency renal nerve ablation" OR "radiofrequency renal denervation" OR "radio-frequency renal denervation" OR "renal ablation" OR "sympathetic renal denervation" OR "renal nerve modulation" OR "kidney denervation" OR "sympathectomy" OR "radiofrequency ablation" OR "radiofrequency ablation" OR “Alcohol Mediated renal denervation”) |
| **#5** | #2 OR #3 |
| **#6** | #1 AND #4 AND #5 |

**Supplementary Table 2:** Summary of secondary outcomes, subgroups, and sensitivity analyses

| Index | Sensitivity or Subgroup analysis | MD and 95% CI | Heterogeneity |
| --- | --- | --- | --- |
| 24h AMSBP | Fixed Common effect model | -2.42 mmHg, 95% CI: -2.74 to -2.11 | I²=91% |
| 24h AMSBP | LOO | -2.63 mmHg, 95% CI: -4.26 to -1.00, P<0.01 | I²=91% |
| 24h AMSBP | SS>100 | -3.41 mmHg, 95% CI: -5.10 to -1.72 | I² = 91% |
| 24h AMDBP | Fixed Common effect model | -1.62 mmHg, 95% CI: -1.84 to -1.41 | I² = 93% |
| 24h AMDBP | LOO | -1.27 mmHg, 95% CI: -2.31 to -0.22, P=0.02 | I² = 93% |
| 24h AMDBP | SS>100 | -2.36 mmHg, 95% CI: -3.75 to -0.97 | I² = 72% |
| Office SBP | Fixed Common effect model | -4.45 mmHg, 95% CI: -4.85 to -4.05 | I² = 89% |
| Office SBP | LOO | -4.26 mmHg, 95% CI: -5.68 to -2.84, P<0.01 | I² = 89% |
| Office SBP | SS>100 | -5.44 mmHg, 95% CI: -6.93 to -3.95 | I² = 81% |
| Office DBP | Fixed Common effect model | - 2.38 mmHg, 95% CI: -2.68 to -2.07 | I² = 96% |
| Office DBP | LOO | -2.15 mmHg, 95% CI: -3.40 to -0.90, P<0.01 | I² = 96% |
| Office DBP | SS>100 | -2.95 mmHg, 95% CI: -4.28 to -1.63 | I² = 66% |
| Home SBP | Fixed Common effect model | -1.78 mmHg, 95% CI: -2.38 to -1.19 | I² = 0% |
| Home SBP | LOO | -1.78 mmHg, 95% CI: -2.38 to -1.19, P<0.01 | I² = 0% |
| Home DBP | Fixed Common effect model | - 0.07 mmHg, 95% CI: -0.29 to 0.42 | I² = 0% |
| Home DBP | LOO | -0.07 mmHg, 95% CI: -0.29 to 0.42, P=0.07 | I² = 0% |
| Nighttime SBP | Fixed Common effect model | -2.01 mmHg,95% CI: -2.51 to -1.51 | I² = 91% |
| Nighttime SBP | LOO | -2.14 mmHg, 95% CI: -4.58 to 0.30, P=0.09 | I² = 91% |
| Nighttime DBP | Fixed Common effect model | - 0.28 mmHg, 95% CI: -0.12 to 0.68 | I² = 86% |
| Nighttime DBP | LOO | -0.81 mmHg, 95% CI: -2.85 to 1.22, P=0.87 | I² = 86% |
| Daytime SBP | Fixed Common effect model | -1.70 mmHg, 95% CI: -2.13 to -1.26 | I² = 80% |
| Daytime SBP | LOO | -3.29 mmHg, 95% CI: -5.43 to -1.15, P<0.01 | I² = 80% |
| Daytime DBP | Fixed Common effect model | -7.92 mmHg, 95% CI: -8.24 to -7.59 | I² = 97% |
| Daytime DBP | LOO | -2.97 mmHg, 95% CI: -5.64 to -0.30, P=0.03 | I² = 97% |
| Anti-HTN Med | Fixed Common effect model | -0.06, 95% CI: -0.16 to 0.03 | I² = 70% |
| Anti-HTN Med | LOO | -0.08,95% CI: -0.25 to 0.10, P=0.38 | I² = 70% |
| Anti-HTN Med | SS>100 | -0.03, 95% CI: -0.38 to 0.32 | I² = 89% |
| Drug Index | Fixed Common effect model | -0.23, 95% CI: -0.33 to -0.12, | I² = 38% |
| Drug Index | LOO | -0.23, 95% CI: -0.33 to -0.12, P<0.01 | I² = 38% |

**Abbreviations:** SS: sample size, LOO: Leave-one-out, SBP: Systolic blood pressure, DBP: Diastolic blood pressure, HTN: hypertension, Med: Medications

**Supplementary Table 3.** The comparison between the drug index and the average of anti-hypertension medication before and after the intervention and follow-up time.

| **Trial** |  | **Drug Index** | | | | | | | | | **Average of anti-HTN medication** | | | | | | | | |
| --- | --- | --- | --- | --- | --- | --- | --- | --- | --- | --- | --- | --- | --- | --- | --- | --- | --- | --- | --- |
|  |  | **Baseline** | | | | **Final** | | | | | **Baseline** | | | | **Final** | | | | |
|  | **FU time** | **RD** | **SD** | **SH** | **SD** | | **RD** | **SD** | **SH** | **SD** | **RD** | **SD** | **SH** | **SD** | | **RD** | **SD** | **SH** | **SD** |
| **RADIANCE II, Azizi, 2023** | **2m** |  |  |  |  | |  |  |  |  |  |  |  |  | | **2.50** | **1.40** | **2.00** | **0.00** |
| **REINFORCE, Weber, 2020** | **8w** |  |  |  |  | |  |  |  |  |  |  |  |  | | **1.80** | **0.80** | **1.60** | **0.60** |
| **REQUIRE, Kario, 2022** | **3m** |  |  |  |  | |  |  |  |  | **4.20** | **1.70** | **3.90** | **1.20** | | **4.10** | **1.20** | **4.20** | **1.30** |
| **ReSET, Mathiassen, 2016 ^3^** | **6m** | **6.90** | **2.70** | **6.80** | **2.50** | | **6.50** | **2.80** | **7.10** | **2.50** | **4.10** | **1.20** | **4.20** | **1.10** | | **4.10** | **1.20** | **4.20** | **1.30** |
| **SPYRAL HTN-ON MED, Kandazari, 2023** | **6m** | **1.43** | **1.19** | **1.38** | **1.04** | | **1.29** | **1.14** | **1.42** | **1.01** | **2.13** | **1.40** | **1.98** | **1.14** | | **1.84** | **1.37** | **2.05** | **1.14** |
| **RADIANCE-HTN SOLO, Azizi, 2019** | **6m** | **0.70** | **1.00** | **0.50** | **0.50** | | **0.50** | **0.50** | **0.70** | **0.60** | **1.20** | **0.70** | **1.20** | **0.80** | | **0.90** | **0-3** | **1.30** | **0-4** |
| **RADIANCE-HTN SOLO, Azizi, 2020** | **12m** | **0.70** | **1.00** | **0.50** | **0.50** | | **0.50** | **0.50** | **0.80** | **0.60** | **1.20** | **0.70** | **1.20** | **0.80** | | **1.00** | **0.80** | **1.20** | **0.80** |
| **SPYRAL ON MED Mahfoud 2023** | **36m** | **1.43** | **1.19** | **1.38** | **1.04** | | **2.04** | **1.16** | **2.19** | **1.18** | **2.13** | **1.40** | **1.98** | **1.14** | | **2.97** | **1.21** | **2.95** | **0.99** |
| **SPYRAL ON MED Kario, 2022** | **36m** | **1.43** | **1.19** | **1.38** | **1.04** | | **2.13** | **1.15** | **2.55** | **2.19** | **2.13** | **1.40** | **1.98** | **1.14** | | **3.03** | **1.20** | **3.05** | **1.16** |
| **Symplicity HTN-3, Bhatt 2014** | **6m** |  |  |  |  | |  |  |  |  | **5.10** | **1.40** | **5.20** | **1.40** | | **5.00** | **1.40** | **5.20** | **1.43** |
| **SMART, Wang, 2024** | **6m** | **9.17** | **7.11** | **9.04** | **6.11** | | **13.16** | **8.35** | **16.03** | **9.74** | **2.76** | **0.85** | **2.79** | **0.85** | | **3.04** | **0.87** | **3.25** | **0.89** |

**Abbreviation**: FU: Follow-up, RD: Renal Denervation, SD: Standard Deviation, SH: Sham Control

**Supplementary Table 4**. Complications of Renal Sympathetic Denervation (RSD) and Sham Procedures

| **Trials** | **Emb + EOD** | | **MASC** | | **PRP>2** | | **AER** | | **SAER** | | **SCVAER** | | **AKI** | | **ACM** | | **MVC** | | **HTN ER** | | **Hypo ER** | | **HHF** | | **CVA** | | **MI** | | **CRR>50%** | | **RAS>70%** | | **PCI** | |
| --- | --- | --- | --- | --- | --- | --- | --- | --- | --- | --- | --- | --- | --- | --- | --- | --- | --- | --- | --- | --- | --- | --- | --- | --- | --- | --- | --- | --- | --- | --- | --- | --- | --- | --- |
|  | **RD** | **SH** | **RD** | **SH** | **RD** | **SH** | **RD** | **RD** | **RD** | **SH** | **RD** | **SH** | **RD** | **SH** | **RD** | **SH** | **RD** | **SH** | **RD** | **SH** | **RD** | **SH** | **RD** | **RD** | **RD** | **SH** | **RD** | **SH** | **RD** | **SH** | **RD** | **SH** | **RD** | **SH** |
| **RADIANCE-HTN TRIO, Azizi, 2021** | **0** | **0** | **1** | **0** | **12** | **0** | **0** | **0** | **0** | **0** | **0** | **0** | **0** | **0** | **1** | **0** | **0** | **0** | **0** | **0** | **0** | **0** | **0** | **0** | **0** | **0** | **1** | **0** | **1** | **0** | **0** | **0** | **0** | **0** |
| **REQUIRE Kario 2022** | **0** | **0** | **0** | **0** | **6** | **6** | **0** | **0** | **0** | **0** | **0** | **0** | **0** | **0** | **0** | **0** | **0** | **0** | **0** | **0** | **0** | **0** | **0** | **0** | **0** | **0** | **0** | **0** | **0** | **0** | **0** | **0** | **0** | **0** |
| **OFF MED Pivotal, Bohm 2020** | **0** | **0** | **0** | **0** | **0** | **0** | **0** | **0** | **0** | **0** | **0** | **0** | **0** | **0** | **0** | **0** | **0** | **0** | **1** | **0** | **0** | **0** | **0** | **0** | **0** | **1** | **0** | **0** | **0** | **0** | **0** | **0** | **0** | **0** |
| **SPYRAL ON MED, Kandazari 2023** | **0** | **0** | **0** | **0** | **0** | **0** | **0** | **0** | **0** | **0** | **0** | **0** | **0** | **0** | **0** | **0** | **0** | **0** | **1** | **0** | **0** | **0** | **0** | **0** | **1** | **0** | **0** | **0** | **0** | **0** | **0** | **0** | **0** | **0** |
| **Wave IV, Schmieder, 2017** | **0** | **0** | **0** | **0** | **0** | **0** | **30** | **26** | **3** | **8** | **0** | **0** | **0** | **0** | **0** | **0** | **0** | **0** | **4** | **2** | **1** | **1** | **0** | **0** | **0** | **0** | **0** | **0** | **0** | **0** | **0** | **0** | **0** | **0** |
| **RADIANCE-HTN SOLO, Azizi, 2020** | **0** | **0** | **0** | **0** | **0** | **0** | **0** | **0** | **0** | **0** | **0** | **0** | **0** | **0** | **0** | **1** | **0** | **0** | **0** | **0** | **0** | **0** | **0** | **0** | **0** | **0** | **0** | **0** | **0** | **0** | **0** | **0** | **0** | **0** |
| **RADIANCE-HTN TRIO, Azizi, 2022** | **0** | **0** | **0** | **0** | **12** | **0** | **0** | **0** | **0** | **0** | **0** | **0** | **0** | **0** | **0** | **0** | **0** | **0** | **0** | **1** | **0** | **0** | **0** | **0** | **0** | **1** | **1** | **1** | **0** | **0** | **0** | **0** | **2** | **1** |
| **SPYRAL ON MED, Mahfoud 2023** | **0** | **0** | **0** | **0** | **0** | **0** | **0** | **0** | **0** | **0** | **0** | **0** | **0** | **0** | **0** | **0** | **0** | **0** | **1** | **0** | **0** | **0** | **0** | **0** | **1** | **0** | **0** | **0** | **0** | **0** | **0** | **0** | **0** | **0** |
| **Symplicity HTN-3, Bhatt 2014** | **1** | **0** | **0** | **0** | **0** | **0** | **0** | **0** | **0** | **0** | **0** | **0** | **0** | **0** | **2** | **1** | **1** | **0** | **9** | **9** | **0** | **0** | **9** | **3** | **4** | **2** | **6** | **3** | **5** | **1** | **1** | **0** | **0** | **0** |
| **TARGET BPI, Kandazari, 2024** | **0** | **0** | **0** | **0** | **0** | **0** | **11** | **6** | **0** | **0** | **0** | **0** | **0** | **0** | **1** | **0** | **1** | **0** | **2** | **2** | **7** | **3** | **0** | **0** | **0** | **0** | **1** | **1** | **0** | **0** | **0** | **0** | **0** | **0** |
| **SMART, Wang 2024** | **0** | **0** | **0** | **0** | **0** | **0** | **75** | **68** | **11** | **8** | **1** | **4** | **0** | **0** | **0** | **0** | **0** | **0** | **0** | **0** | **0** | **0** | **0** | **0** | **0** | **0** | **0** | **0** | **0** | **0** | **1** | **0** | **0** | **0** |
| **IBERIS**  **Jiang, X,2024** | **0** | **0** | **0** | **0** | **0** | **0** | **0** | **0** | **0** | **0** | **0** | **0** | **0** | **0** | **0** | **0** | **0** | **0** | **0** | **0** | **0** | **0** | **0** | **0** | **0** | **0** | **0** | **0** | **0** | **0** | **0** | **0** | **2** | **1** |

**Abbreviations:** EMB+EOD: Embolic event and end-organ damage, MASC: Major access site complications, PRP>2D: Procedure-related pain>2 days, AER: Adverse event rate, SAER: Severe adverse event rate, SCVAER: Severe cerebrovascular adverse event rate, AKI: Acute kidney injury, ACM: All-cause mortality, MVC: Major vascular complications, HTNER: Hypertension emergency, HypoER: Hypotension emergency, AKI: Acute kidney injury, HHF: Hospitalization for heart failure, CVA: cerebrovascular accidents, MI: Myocardial infarction, CRR: Creatinine rise, RAS: Renal artery stenosis, PCI: Percutaneous coronary intervention.

**Supplementary Table 5:** A comparison of treatment effects on blood pressure outcomes between random effects and fixed effect models

| Outcome | Random-Effects Model | Fixed-Effect Model |
| --- | --- | --- |
| 24-hour SBP | -2.63 mmHg, 95% CI: -4.26 to -1.00, P<0.01 | -2.42 mmHg, 95% CI: -2.74 to -2.11 |
| 24-hour DBP | -1.22 [-2.29; -0.15] | -1.62[-1.84; -1.40] |
| Office SBP | -4.26 mmHg, 95% CI: -5.68 to -2.84, P<0.01 | -4.45 mmHg, 95% CI: -4.85 to -4.05 |
| Office DBP | -2.15 mmHg, 95% CI: -3.40 to -0.90, P<0.01 | - 2.38 mmHg, 95% CI: -2.68 to -2.07 |
| Home SBP | -1.78 [-2.38; -1.19] | -1.78 [-2.38; -1.19] |
| Home DBP | 0.07 [-0.29; 0.42] | 0.07 [-0.29; 0.42] |
| Night SBP | -2.14 mmHg, 95% CI: -4.58 to 0.30, P=0.09 | -2.01 mmHg,95% CI: -2.51 to -1.51 |
| Night DBP | -0.81 mmHg, 95% CI: -2.85 to 1.22, P=0.87 | - 0.28 mmHg, 95% CI: -0.12 to 0.68 |
| Day SBP | -3.29 mmHg, 95% CI: -5.43 to -1.15, P<0.01 | -1.70 mmHg, 95% CI: -2.13 to -1.26 |
| Day DBP | -2.97 mmHg, 95% CI: -5.64 to -0.30, P=0.03 | -7.92 [-8.24; -7.59] |
